# Combination Chemo‐Immunotherapy for Pancreatic Cancer Using the Immunogenic Effects of an Irinotecan Silicasome Nanocarrier Plus Anti‐PD‐1

**DOI:** 10.1002/advs.202002147

**Published:** 2021-01-27

**Authors:** Xiangsheng Liu, Jinhong Jiang, Yu‐Pei Liao, Ivanna Tang, Emily Zheng, Waveley Qiu, Matthew Lin, Xiang Wang, Ying Ji, Kuo‐Ching Mei, Qi Liu, Chong Hyun Chang, Zev A. Wainberg, Andre E. Nel, Huan Meng

**Affiliations:** ^1^ Division of Nanomedicine Department of Medicine University of California Los Angeles CA 90095 USA; ^2^ California NanoSystems Institute University of California Los Angeles CA 90095 USA; ^3^ Division of Hematology Oncology Department of Medicine University of California Los Angeles CA 90095 USA; ^4^ Present address: The Cancer Hospital of the University of Chinese Academy of Sciences Institute of Basic Medicine and Cancer (IBMC) Chinese Academy of Sciences Hangzhou Zhejiang 310022 China

**Keywords:** autophagy, chemo‐immunotherapy, irinotecan silicasome, pancreatic cancer, PD‐1/PD‐L1 axis

## Abstract

There is an urgent need to develop new life‐prolonging therapy for pancreatic ductal adenocarcinoma (PDAC). It is demonstrated that improved irinotecan delivery by a lipid bilayer coated mesoporous silica nanoparticle, also known as a silicasome, can improve PDAC survival through a chemo‐immunotherapy response in an orthotopic Kras‐dependent pancreatic cancer model. This discovery is premised on the weak‐basic properties of irinotecan, which neutralizes the acidic lysosomal pH in PDAC cells. This effect triggers a linked downstream cascade of events that include autophagy inhibition, endoplasmic reticulum stress, immunogenic cell death (ICD), and programmed death‐ligand 1 (PD‐L1) expression. ICD is characterized by calreticulin expression and high‐mobility group box 1 (HMGB1) release in dying Kras‐induced pancreatic cancer (KPC) cells, which is demonstrated in a vaccination experiment to prevent KPC tumor growth on the contralateral site. The improved delivery of irinotecan by the silicasome is accompanied by robust antitumor immunity, which can be synergistically enhanced by anti‐PD‐1 in the orthotopic model. Immunophenotyping confirms the expression of calreticulin, HMGB1, PD‐L1, and an autophagy marker, in addition to perforin and granzyme B deposition. The chemo‐immunotherapy response elicited by the silicasome is more robust than free or a liposomal drug, Onivyde. The silicasome plus anti‐PD‐1 leads to significantly enhanced survival improvement, and is far superior to anti‐PD‐1 plus either free irinotecan or Onivyde.

## Graphical abstract

Improved irinotecan delivery by silicasome can improve pancreatic ductal adenocarcinoma (PDAC) survival through a chemo‐immunotherapy response when combined with anti‐PD‐1 in an orthotopic Kras‐dependent PDAC model. This discovery is premised on the weak‐basic properties of irinotecan, which neutralizes the acidic lysosomal pH and triggers a linked downstream cascade of events that include autophagy inhibition, endoplasmic reticulum stress, immunogenic cell death, and PD‐L1 expression.

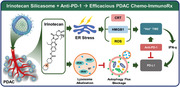

## 1 Introduction

**Figure 1:**
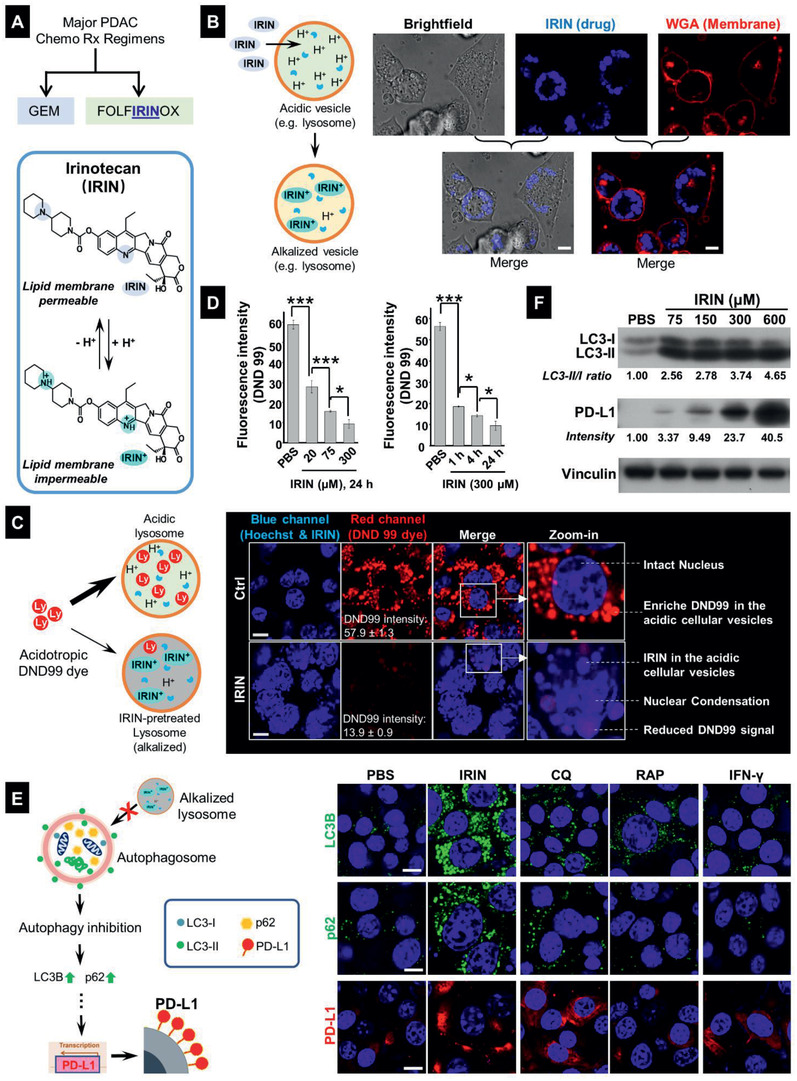
The alkalizing effect of free IRIN leads to autophagy inhibition and upregulation of PD‐L1 expression in KPC cells. A) IRIN, a major PDAC cancer drug, is a weak base that can be protonated in an acidic environment. B) Confocal microscopy to demonstrate the localization of the amphiphilic drug, in organelles close to the surface membrane of KPC cells, exposed to 300 × 10^−6^ m IRIN for 24 h. The drug exhibits blue fluorescence at an excitation wavelength of 405 nm. The cell membrane was stained by Alexa Fluor 594 conjugated WGA (red). Bar: 10 µm. C) Representative confocal microscopy to demonstrate that IRIN (300 × 10^−6^ m, 24 h) could neutralize the acidic pH of lysosomes that were stained by the red fluorescent acidotropic dye, DND 99 Lysotracker. Alkalization of these organelles by IRIN resulted in a sharp reduction of DND 99 fluorescence, which is overtaken by the blue fluorescence of the drug in the same compartment. Costaining with Hoechst 33342 showed the presence of nuclear condensation in IRIN‐treated cells. Bar: 10 µm. D) Dose‐ and time‐dependent study of the lysosomal alkalization effect of free IRIN at the indicated concentrations (left) and incubation time periods (right). Image J software analysis was used to quantify the change in DND 99 fluorescence intensity. Data represents mean ± SD, *n* = 3. *p < 0.05; ***p < 0.001 (1‐way ANOVA followed by a Tukey's test). The corresponding confocal images appear in Figure S2 (Supporting Information). E) IF staining of LC3B, p62, and PD‐L1 in KPC cells exposed to IRIN (300 × 10^−6^ m), CQ (32 × 10^−6^ m), RAP (100 × 10^−9^ m), or IFN‐*γ* (10 ng mL^−1^) for 24 h. Bar is 10 µm. F) Immunoblotting of LC3 and PD‐L1 in KPC lysates following cellular treatment with IRIN at the indicated concentrations for 24 h. Densitometric analysis was performed by ImageJ software and the fold of intensity was normalized to vinculin.

Pancreatic ductal adenocarcinoma (PDAC) is a lethal disease with a 5‐year survival rate of ≈8%.^[^ ^1^ ^]^ According the guidelines of the American Cancer Society, the best available chemotherapy options for advanced disease are treatment with gemcitabine (GEM)/nab‐paclitaxel or a four‐drug regimen, known as FOLFIRINOX (folinic acid, 5‐fluorouracil, irinotecan, oxaliplatin) (**Figure** **1**A).^[^ ^2^ ^]^ The FOLFIRINOX regimen was modified in 2018 to allow resected PDAC patients to be treated with a reduced irinotecan dose (150 instead of 180 mg m^−2^) for 24 weeks.^[^ ^2d^ ^]^ The outcome was encouraging in the patients who had been resected, demonstrating a median overall survival of 54.4 months in the modified FOLFIRINOX group versus 35.0 months in the control arm (GEM monotherapy) (*p* < 0.01).^[^ ^2a,d^ ^]^ While more clinical validation studies are ongoing, it is suggested that the modified regimen is gaining support. Moreover, recent studies of liposomal irinotecan (Onivyde) have led to its approval in metastatic pancreas cancer in patients having progressed on gemcitabine.^[^ ^3^ ^]^ This provided the first evidence that a liposomal formulation of irinotecan in pancreatic cancer has clinical utility.

In addition to new ways in which chemotherapy is being used, we are drawing on the game‐changing advances that have been introduced immune checkpoint inhibitors (ICIs) to treat cancers such as melanoma, renal, and lung cancer.^[^ ^4^ ^]^ However, there has been little success in the use of immune checkpoint blocking antibodies in PDAC.^[^ ^5^ ^]^ While anti‐PD‐1 antibody (Keytruda) was approved for PDAC patients with rare genetic mutations (i.e., microsatellite instability or mismatch repair deficiency), this treatment option only impacts less than 1% of cases.^[^ ^6^ ^]^ Although the exact rate of PD‐L1 expression in PDAC is controversial, several studies have suggested that this biomarker is expressed in only ∼10% of cases.^[^ ^7^ ^]^ However, higher rates have also been reported,^[^ ^8^ ^]^ which is indicative of the heterogeneous PDAC immune landscape as well as the lack of consensus in how to perform PD‐L1 quantification.^[^ ^9^ ^]^ Nevertheless, it is generally agreed upon that the general lack of expression of immune checkpoint receptors is an important reason for the poor response to PD‐1/PD‐L1 blockade in this disease.^[^ ^7^, ^9^ ^]^ Other factors, such as low tumor immunogenicity (“cold tumors”), low mutational load, poor drug access, accumulation of regulatory T‐cells (Tregs), stroma‐mediated immunosuppression, and regional expression of a host of additional immune escape pathways also contribute to failed immunotherapy in PDAC.^[^ ^10^ ^]^

In spite of the poor response to chemotherapy, it has become popular for many solid tumors, including PDAC, to address the immune‐suppressive tumor microenvironment (TME) by introducing combination therapy in an attempt to augment the ICI responsiveness.^[^ ^5^, ^11^ ^]^ A promising approach is to utilize the immunogenic properties of certain chemotherapeutic agents, such as anthracyclines (e.g., doxorubicin, DOX) or oxaliplatin (OX), capable of increasing the recruitment of cytotoxic T cells (CTL) to the “cold” TME, i.e., switching its immune status to “hot.”^[^ ^12^ ^]^ This immunogenic effect is dependent on off‐target effects of the chemo agents on cellular sites such as the endoplasmic reticulum (ER), where the generation of cell stress responses can trigger translocation of calreticulin (CRT) to the dying tumor cell surface; CRT serves as an “eat me” signal for cancer cell engulfment by antigen‐presenting cells (APC).^[^ ^12^, ^13^ ^]^ This allows APC display of endogenous tumor‐associated antigens to naïve T‐cells.^[^ ^12^, ^13^ ^]^ In addition, disintegration of the nuclei of dying tumor cells, leads to the release of high‐mobility group box 1 (HMGB1) protein, which acts as an adjuvant by engaging TLR4 receptors on APC.^[^ ^12^, ^13^ ^]^ There is also a critical contribution to the immunogenic effects of above chemo agents through induction of autophagy and ATP release.^[^ ^12^, ^14^ ^]^ The collective effect of CRT, HMGB1 and autophagy is to generate immunogenic cell death (ICD) responses by above chemo agents to provide an endogenous vaccination effect that can be used to complement the chemotherapy response. Moreover, ICD leads to the activation and recruitment of cytotoxic T‐cell‐lymphocytes (CTL), the killing effect of which can be boosted by the use of checkpoint blocking antibodies.^[^ ^12c–e,g^ ^]^ In PDAC, for example, it has been demonstrated that oxaliplatin is capable of triggering immunogenic effects in human PANC‐1 and murine Pan02 models.^[^ ^12b^ ^]^ However, the deliberate implementation of chemotherapeutic agents to induce immune responses has not as yet been accomplished as a reproducible treatment option in the clinic because it is difficult to control the delivery of ICD stimuli, which is a particular challenge for PDAC in light of the restricted drug access to the tumor site as a result of the dysplastic stroma.^[^ ^15^ ^]^

We have previously demonstrated improved irinotecan (IRIN) delivery by mesoporous silica nanoparticles (MSNP), coated with a lipid bilayer, in a robust treatment‐resistant Kras‐induced pancreatic cancer (KPC) model,^[^ ^16^ ^]^ derived from a spontaneous Kras^LSL‐G12D/+^; Trp53^LSL‐R172H/+^; Pdx‐1‐Cre (KPC) tumor.^[^ ^17^ ^]^ We also refer to the MSNPs carrier as a “silicasome.”^[^ ^16^ ^]^ While effective for improving the chemotherapy response in the orthotopic KPC model, the experimentation did not explore the immunogenic effects of IRIN, which until now have been labeled as “nondetermined.”^[^ ^12e^ ^]^ The possibility that an immunogenic effect must be entertained emerged from studies on the KPC cell line, which demonstrated that IRIN could induce CRT expression and HMGB1 release that curiously was combined with autophagy inhibition instead of autophagy flux simulation, as seen in the conventional ICD model.^[^ ^12^, ^14^ ^]^ This prompted extensive mechanistic investigation into the biological effects of IRIN to explain its immunogenic effects, including whether these responses could be used to initiate immunotherapy in vivo. Our results will delineate that an additional dimension of the IRIN treatment response involves the neutralizing effect of the free or encapsulated drug on lysosomal pH, which is associated with autophagy inhibition and triggering of an ICD response that is dependent on primary ER stress. This allowed us to assign IRIN as a “Type II” ICD inducer, an ICD stimulus that has been described from the perspective of photodynamic therapy.^[^ ^12^, ^14^ ^]^ Moreover, we also show that the autophagy inhibition is associated with increased PD‐L1 expression on KPC cells. These findings provided the basis for studying the chemo‐immunotherapy response to IRIN‐delivery silicasomes in an orthotopic KPC model. We also asked whether the effect could be combined with the delivery of anti‐PD‐1 antibodies.

## 2 Results

### 2.1 IRIN Leads to Lysosomal Alkalization, Which is Linked to Autophagy Inhibition and PD‐L1 Overexpression in KPC Cells

IRIN is a weak base (pKa = 8.1) that can be readily protonated in an acidic environment.^[^ ^18^ ^]^ In fact, we make use of this property for remote loading of IRIN into the silicasome carrier (Figure S1, Box 2, in the Supporting Information). This is premised on the principle that the nonprotonated, amphiphilic drug, is capable of diffusing across the coated lipid bilayer, where its protonation by an encapsulated trapping agent (triethylammonium sucrose octasulfate) leads to the generation of hydrophilic IRIN, which is incapable of back‐diffusion across the lipid bilayer.^[^ ^16a^ ^]^ The ability to achieve drug compartmentalization across an artificial lipid bilayer also prompted us to ask whether IRIN can cross cell membranes and become entrapped in acidifying cellular compartments? Utilizing the fluorescent (blue) properties of IRIN, it was possible to demonstrate in a confocal study that the drug was taken up in a vesicular compartment that localizes close to the wheat germ agglutinin (WGA) stained surface membrane in KPC cells (Figure 1B). To confirm that this constitutes an acidifying compartment, a weak‐basic acidotropic dye, Lysotracker Red DND 99, was used to determine the impact of IRIN on the red fluorescence in a confocal microscopy experiment^[^ ^19^ ^]^ (Figure 1C). Thus, while DND 99 could be seen to localize in the lysosomal compartment of untreated KPC cells (Figure 1C, upper right panel), there was a sharp reduction in the dye's red fluorescence intensity in cells that were prior treated with IRIN (Figure 1C, lower right panel). Image J software was used for quantifying the shift in DND 99 fluorescence intensity, allowing us to demonstrate that IRIN treatment could significantly reduce the relative staining intensity from 57.9 ± 1.3 to 13.9 ± 0.9 in KPC cells. This allowed the blue drug fluorescence to be observed, in addition to the appearance of nuclear condensation in the dying cells (Figure 1C). Noteworthy, the IRIN treatment effect was both dose‐ (Figure 1D, left) and time‐dependent (Figure 1D, right); the corresponding confocal images appear in Figure S2A,B. The alkalizing effect of IRIN was duplicated by chloroquine (CQ), which is a frequently used weak‐base lysosomal alkalizing agent in cancer cell biology (Figure S2C, Supporting Information).^[^ ^20^ ^]^ The effect of the encapsulated drug will be discussed later.

In addition to the role of the acidic pH in the destruction of lysosomal content, lysosomal acidification is also important for the fusion of the organelle with the autophagosome.^[^ ^20^, ^21^ ^]^ Thus, we were interested to determine if IRIN can interfere in autophagy flux, as previously demonstrated, through the use of CQ or gene deletion of the proton‐generating V‐ATPase subunit of the lysosome.^[^ ^22^ ^]^ The methodology for demonstrating autophagy inhibition is to show the presence of LC3B complexes, which are involved in the formation of the autophagosome, as well as the accumulation of the sensor protein, p62/SQSTM1. The p62/SQSTM1 detects toxic cellular waste products and is removed and destroyed with the waste products in the lysosome.^[^ ^20^, ^21^, ^23^ ^]^ This was accomplished by the performance of confocal microscopy to demonstrate the intracellular appearance of immunofluorescence (IF) stained LC3B and p62/SQSTM1 complexes, as shown in Figure 1E. Confocal viewing demonstrated that IRIN treatment leads to the contemporaneous appearance of fluorescent LC3B puncta as well as p62 protein complexes in KPC cells (Figure 1E, right panel). In order to confirm that dual fluorescence staining discerned autophagy inhibition, we also used CQ to demonstrate the appearance of similar immunofluorescence features (Figure 1E). In contrast, rapamycin (RAP), which functions as an autophagy inducer, resulted in LC3B assembly without p62 accumulation.^[^ ^20^, ^21^, ^23^ ^]^ The assembly of LC3B complexes were confirmed in an immunoblotting assay, which demonstrated a dose‐dependent increase in the ratio of the LC3‐II to LC3‐I expression during IRIN treatment (Figure 1F).

Of specific importance to the objective of addressing IRIN immunogenicity, it has recently been demonstrated that the accumulation of p62/SQSTM1 during pharmacological disruption of autophagy can trigger PD‐L1 expression in gastric cancer cells.^[^ ^23^ ^]^ Not only did we demonstrate IRIN treatment can induce robust cell surface expression of PD‐L1 in KPC cells during the performance of confocal microscopy (Figure 1E, bottom right panel), but also observed the same effect during immunoblotting (Figure 1F). These results were in in agreement with the effect of CQ or cellular treatment with IFN‐*γ*, a robust inducer of the PD‐L1 promoter^[^ ^24^ ^]^ (Figure 1E, bottom right panel). Noteworthy, these responses were dose‐ and time‐dependent, as shown in the immunoblotting (Figure 1F) and confocal experiments (Figure S3A,B, Supporting Information). A possible mechanism to explain PD‐L1 expression by p62/SQSTM1 in gastric cancer has been the demonstration of NF‐*κ*B activation, which impacts the PD‐L1 promoter.^[^ ^23^ ^]^ We confirmed that that IRIN treatment can induce the phosphorylation of the p65 (p‐p65) subunit of NF‐*κ*B in KPC cells (Figure S4, Supporting Information). In contrast to IRIN, oxaliplatin (OX), a potent nonbasic PDAC chemo agent induced autophagy (Figure S5, Supporting Information) but failed to upregulate PD‐L1 expression in KPC cells (Figure S6, Supporting Information).

### 2.2 The Autophagosomal Inhibitory Effect of IRIN is Accompanied by Endoplasmic Reticulum (ER) Stress, An Inducer of Immunogenic Cell Death Pathways

**Figure 2:**
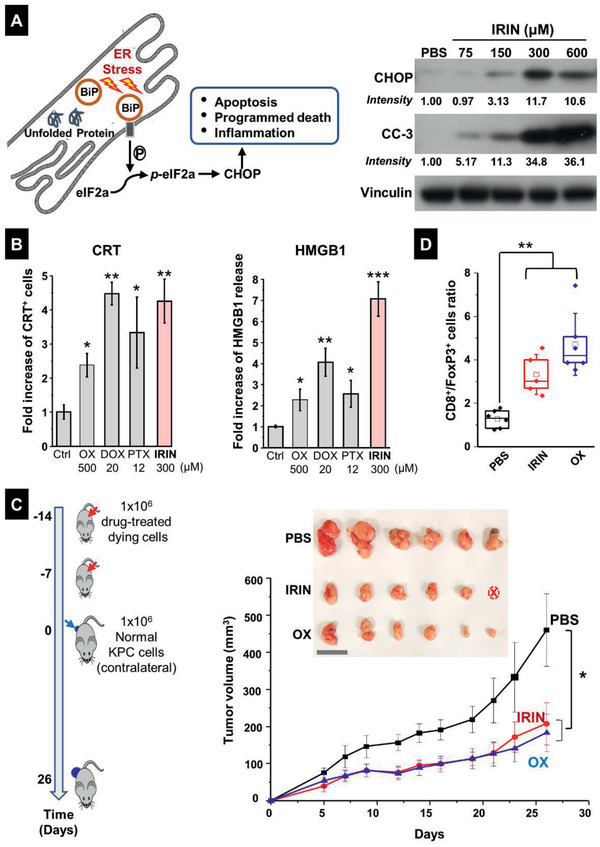
Assessment of ER stress responses induced by free IRIN in KPC cells. A) Left: Simplified schematic to show the unfolded protein stress response in the ER. Right: immunoblotting to show the expression of the ER stress response marker CHOP and cleaved caspase 3 (CC‐3) in KPC cells treated with IRIN for 24 h. B) CRT expression was assessed by flow cytometry (left panel), while HMGB1 release was determined by ELSLA (right panel) in KPC cells exposed to OX (500 × 10^−6^ m), IRIN (300 × 10^−6^ m), DOX (20 × 10^−6^ m), and PTX (12 × 10^−6^ m) for 24 h. Data are expressed as mean ± SD. *n* = 3. *p < 0.05; **p < 0.01; ***p < 0.001 compared to PBS control (Student's *t*‐test). C) Vaccination experiment in a PDAC mouse model. Left: The schematic shows execution of the vaccination study through subcutaneous injection of dying KPC cells treated with IRIN or OX, followed by rechallenge with untreated KPC cells. As a negative control in the vaccination experiment, mice were treated with PBS only, without cellular debris. Right: Tumor growth curves of normal KPC cells, injected in the opposite flank of the vaccinated animals. Data are expressed as mean ± SEM. *n* = 6. Insert: Photography of the harvested tumors collected for each group. Note that there was one tumor free animal in the IRIN group (labeled as “ⓧ”). Bar: 2 cm. D) Quantitative assessment of CD8^+^/FoxP3^+^ cell ratios by IHC analysis. *p < 0.05; **p < 0.01 (1‐way ANOVA followed by a Tukey's test).

The ablation of autophagy has been linked to the generation of ER stress and the generation of immunogenic cell death in cancer cells during hypericin‐mediated photodynamic therapy (Hyp‐PDT).^[^ ^25^ ^]^ Not only is ER stress a common feature of cellular damage, but has also been used to distinguish between a “Type I” ICD response that primarily targets the nucleus, with secondary impact on the ER, as compared to a “Type II” ICD response in which ER stress is the primary event that secondarily leads to cell death and nuclear involvement.^[^ ^13^, ^14^, ^26^ ^]^ Thus, while most chemotherapeutics (e.g., DOX and OX) engaged in ICD effects have been characterized as “Type I” ICD inducers, a few novel platinum agents (e.g., Pt–N‐heterocyclic carbene) and physicochemical stimuli (e.g., Hyp‐PDT) are primarily ER stress inducer with secondary effects on nuclear damage and apoptosis.^[^ ^12^, ^27^ ^]^ The basis of the hypericin‐induced effect on the ER has been shown to involve reactive oxygen species (ROS) production that leads to ER‐associated proteotoxicity, also known as an unfolded protein response.^[^ ^13^, ^14^, ^26^ ^]^ We focused on unfolded protein response, which leads to the phosphorylation of the eukaryotic initiation factor (eIF2*α*) that is responsible for transcriptional activation of the CCAAT‐enhancer‐binding protein homologous (CHOP) protein^[^ ^13^, ^28^ ^]^ (**Figure** **2**A, left panel). CHOP, in turn, is capable of inducing apoptotic cell death through the generation of immunological danger signals that promote antitumor immunity.^[^ ^13^, ^28^ ^]^

To determine the impact of IRIN on CHOP expression in KPC cells, immunoblotting analysis was used to show a sizable and dose‐dependent increase in CHOP expression (Figure 2A, right panel). Moreover, this response was accompanied by increased expression of cleaved caspase‐3 (CC‐3), indicative of a linked apoptosis event. In order to assess the involvement of ROS generation, fluorescence microscopy was used to assess the impact of IRIN on “total ROS” production in KPC cells, using an Abcam kit.^[^ ^29^ ^]^ OX, a Type I ICD inducer, as well tunicamycin (TUN), an ER stress generating antibiotic, served as controls (Figure S7, Supporting Information). The data showed robust generation ROS production in IRIN treated KPC cells (Figure S7, Supporting Information). It has previously been shown that the link among ROS production, ER stress and cell death involves the triggering of intracellular Ca^2+^ flux.^[^ ^28^, ^30^ ^]^ In order to assess intracellular Ca^2+^ release, confocal microscopy was used to perform a Fluo‐4 AM assay^[^ ^31^ ^]^ in KPC cells. IRIN treatment led to the highest level of Ca^2+^ release compared to OX and TUN (Figure S8, Supporting Information). All considered, these data indicate that IRIN exerts a robust ER stress response in KPC cells, which distinguishes it from the ICD effect of OX.

### 2.3 IRIN Induces an Immunogenic Response in KPC Cells that Leads to a Successful Vaccination Outcome In Vivo

ICD responses are characterized by the induction of CRT translocation to the dying tumor cell surface, where it serves as an “eat‐me” signal for tumor cell antigen presentation by dendritic cells.^[^ ^13^ ^]^ Cell death is also associated with the release of the chromatin protein, HMGB1, from the damaged cell nuclei.^[^ ^13^ ^]^ IRIN was compared to OX, DOX, and paclitaxel (PTX) in CRT and HGMB1 assays in KPC cells.^[^ ^12^, ^14^, ^27^ ^]^ Utilizing flow cytometry to assess CRT expression on the KPC surface and an enzyme‐linked immunosorbent assay (ELISA) for HMGB1 release, it was possible to demonstrate that IRIN is a strong inducer of both responses (Figure 2B). Moreover, we also confirmed that the CRT response was dose‐dependent (Figure S9A,B, Supporting Information). Extracellular ATP release is often described as the third component of a typical Type I ICD response.^[^ ^14^, ^25^ ^]^ However, it was of interest that we could not demonstrate an increase in ATP release by IRIN, which is in agreement with the induction of a Type II response by Hyp‐PDT^[^ ^25^ ^]^ and previous demonstration that autophagy inhibition is accompanied by ATP consumption.^[^ ^32^ ^]^ Thus, in order to definitively demonstrate that IRIN induces an immunogenic cell response, we made use of an in vivo vaccination experiment as the gold standard for demonstrating assess the response outcome, as per the Consensus Guidelines for detection of ICD.^[^ ^33^ ^]^

A vaccination experiment was performed to determine if the subcutaneous injection of dying KPC cells into the flank of syngeneic B6129SF1/J mice on two occasions could impact the growth of live KPC tumor cells injected on the opposite flank (Figure 2C, left panel).^[^ ^12c,f^ ^]^ A comparison of the vaccination response to KPC cells, treated with 300 × 10^−6^ m IRIN or 500 × 10^−6^ m OX, demonstrated that the generation of cell death by IRIN was comparable to the effect of OX, both of which improved the shrinkage of the tumor on the opposite flank significantly (*p* < 0.05) compared to phosphate‐buffered saline (PBS) control (Figure 2C, right panel). Moreover, tumor harvesting on day 26, followed by collection of bright field pictures, confirmed the growth inhibitory effect of the chemo agents, including total tumor disappearance in one IRIN‐treated animal (right panel). The harvested tumor tissues were also used to conduct immunohistochemistry (IHC) analysis for the expression of the CD8^+^ marker for cytotoxic T‐cells and FoxP3^+^ for Treg cells (Figure S10, Supporting Information). This showed that while there was a slight increase in CD8^+^ staining number in response to OX (but not IRIN), both agents significantly (*p* < 0.01) increased the CD8^+^/FoxP3+ ratio in the quantitative response assessment (Figure 2D).

### 2.4 Encapsulated IRIN Induces Lysosomal Alkalization, Autophagy Inhibition, and PD‐L1 Expression in PDAC Cells

**Figure 3:**
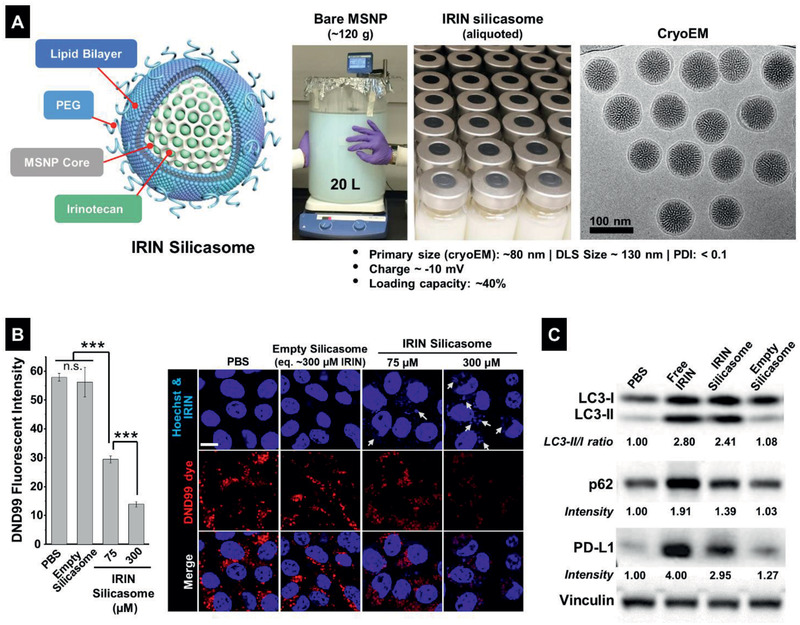
Silicasome synthesis and assessment of lysosomal pH, ER stress and autophagy in KPC cells, using encapsulated IRIN. A) Schematic to explain large batch synthesis and characterization of the silicasome nanocarrier in this study. The carrier is comprised of a MSNP core, which contains a large packaging space for IRIN loading, and encapsulated by a lipid bilayer (comprised of DSPC: Cholesterol: PE‐PEG_2K_ at 3:2:0.15 molar ratio). The fully synthesized carrier was dispensed into vials containing 50 mg IRIN/container. CryoEM was undertaken to show particle morphology, in addition to characterization of size, charge, and IRIN loading capacity, as shown. B). KPC cells were treated with the IRIN silicasome at indicated concentrations for 24 h. Empty silicasomes (equivalent to 300 × 10^−6^ m IRIN) were included as control. The lysosome alkalizing effect was studied with the DND99 dye, similar to Figure 1C. C) KPC cells were treated by free IRIN and IRIN silicasome at drug concentration of 300 × 10^−6^ m for 24 h. Empty silicasomes were used as control. The treated cells were used for further analysis by LC3‐II/I, p62, and PD‐L1 immunoblotting, as described in Figure 1F. Image J software was used to quantify the fluorescent intensity and band density. Data represent mean ± SD, *n* = 3. ***p < 0.001 (1‐way ANOVA followed by a Tukey's test).

Although free IRIN is quite effective for triggering a series of linked effects, where lysosomal alkalization, autophagy inhibition and the generation of immunogenic effects can be accomplished in KPC cells, the in vivo efficacy of the drug is considerably impaired due to the desmoplastic PDAC stroma and interference in vascular access.^[^ ^34^ ^]^ For this reason, a liposomal carrier, Onivyde, was established to address the IRIN delivery problem and to reduce the serious side effects resulting from systemic drug administration.^[^ ^35^ ^]^ While effective, IRIN leakage from the liposome is still responsible for significant side effects, leading to receiving a black box safety warning from the FDA.^[^ ^3^ ^]^ To further improve IRIN delivery and safety profile, we have previously established a silicasome that have the advantage of improved stability of the lipid bilayer, decreased systemic leakage and toxicity and improved IRIN loading compared to the liposome.^[^ ^16c^ ^]^ A new batch of the silicasome formulation was synthesized under GLP conditions, and an aliquot was used to perform physicochemical characterization of the nanocarrier, as demonstrated in **Figure** **3**A.^[^ ^16c^ ^]^ This demonstrated the presence of uniform particles size of ≈130 nm, a slight negative charge, and a drug loading capacity of ∼40 wt%.

In order to assess the nanocarrier impact on lysosomal alkalization, KPC cells were incubated with the silicasomes to deliver IRIN concentrations of 75 × 10^−6^ and 300 × 10^−6^ m (Figure 3B). While at the lower drug dose, the silicasome was capable of reducing the DND 99 signal by ∼50% and by ∼85% at 300 × 10^−6^ m. Empty silicasomes had no effect on the alkalization. Immunoblotting assessment of LC3, p62, and PD‐L1 expression confirmed the ability of the encapsulated drug to increase the LC3‐II/I ratio, p62 accumulation and PD‐L1 expression, similar to free drug (Figure 3C). These effects were also confirmed by confocal microscopy (Figure S11, Supporting Information). Moreover, IRIN also induced intracellular Ca^2+^ flux, ROS production and CHOP expression in KPC cells in a dose‐dependent fashion (Figure S12, Supporting Information). For example, use of the silicasome, to deliver the equivalent of a 300 × 10^−6^ m drug dose over 48 h, induced an 11.2‐fold increase in CHOP expression compared to the control (Figure S12C, Supporting Information). In addition to the profiling of the murine cell line, we also confirmed the ability of IRIN silicasome, to induce lysosome alkalization, autophagy inhibition and PD‐L1 expression in the frequently used human PDAC cell line, PANC‐1 (Figure S13, Supporting Information).

### 2.5 In Vivo Efficacy of the IRIN Silicasome for Inducing a Survival Effect in the Orthotopic KPC Model, Premised on Generation of an Immune Response that is Boosted by Anti‐PD‐1 Antibody

**Figure 4:**
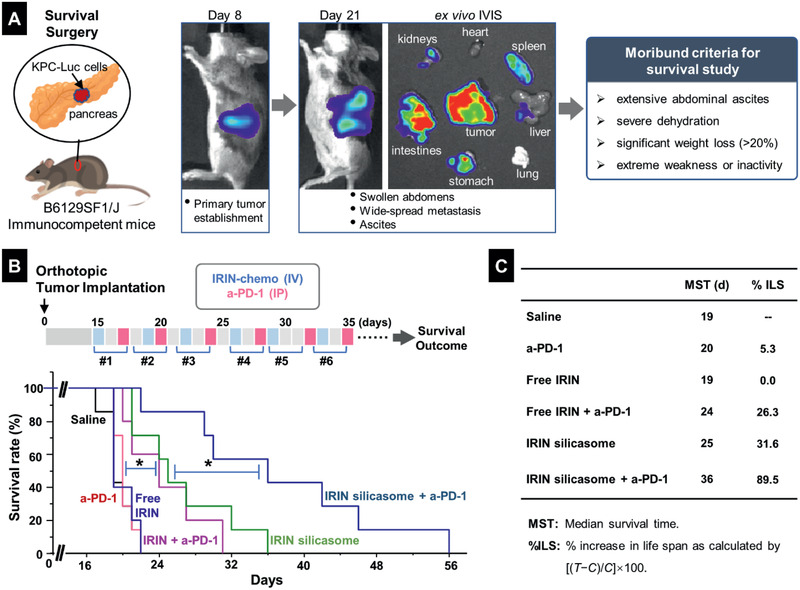
Animal survival study in an orthotopic KPC model, treated with an IRIN silicasome plus anti‐PD‐1 antibody. A) Explanation of the KPC model, including orthotopic implant in the pancreas and technical development of the primary tumor and metastases that can be followed by IVIS imaging. Animals were sacrificed according to the establishedmoribund criteria. B) Details of the survival experiment in tumor‐bearing mice (*n* = 5–7), which were treated with free IRIN or the silicasome at an IRIN dose equivalent of 40 mg kg^−1^ IV every 3 or 4 days, with or without IP administration of 100 µg anti‐PD‐1 antibody, for a total of six administrations. Please notice that the antibody was administered two days after IRIN injection. Saline and anti‐PD‐1 alone were used as controls. Kaplan–Meier plots were used to display the survival rate of the different animal groups (*p < 0.05, Log Rank test). C) Summary of the median survival time (MST) and percentage of increase in life span (%ILS) for each group.

To establish the feasibility to trigger a chemo‐immunotherapy response in an orthotopic KPC model in response to treatment with the IRIN‐silicasome, luciferase‐transfected KPC cells were implanted in the pancreatic tail of immunocompetent B6129SF1/J mice, as previously described and explained in **Figure** **4**A.^[^ ^16^, ^17^ ^]^ We also hypothesized that the accompanying expression of PD‐L1 on KPC cells could allow the immunogenic response to be boosted by coadministering a checkpoint blocking anti‐PD‐1 antibody. The first was a survival experiment in which we compared the effect of free to encapsulated IRIN in the absence or presence of anti‐PD‐1 treatment. Orthotopic KPC tumor‐bearing mice were injected IV with a free or encapsulated IRIN dose of 40 mg kg^−1^ every 3 or 4 days on 6 occasions (Figure 4B, upper panel, blue squares). The treatment was compared to free drug alone or in combination therapy with anti‐PD‐1 antibody, which was injected intraperitoneal (IP) at 100 µg per mouse 2 days after IRIN administration (pink squares). Additional controls included saline injections or mice receiving anti‐PD‐1 antibody alone. Animals were monitored daily until reaching moribund status (Figure 4A) or spontaneous death. This allowed us to generate Kaplan–Meier plots, which were statistically ranked by Log Rank testing (Mantel‐Cox) using GraphPad Prism 7.00 software.^[^ ^16^, ^36^ ^]^ The results demonstrated a significant improvement in the survival (*p* < 0.01) of silicasome‐treated animals compared to free drug or anti‐PD‐1 alone (Figure 4B). Free drug or anti‐PD‐1 had no survival benefits compared to the saline control. While the combined effect of free IRIN plus anti‐PD‐1 was significantly improved compared to monotherapy (*p* < 0.05), the best survival was obtained with the IRIN silicasome plus anti‐PD‐1, which was significantly better than IRIN silicasome alone (*p* < 0.05) or use of free IRIN plus anti‐PD‐1 (*p* < 0.05) (Figure 4B). The survival data was also used to calculate “median survival time” (MST) and percent increase in life span (%ILS) versus saline; this is a frequently used index in preclinical survival studies.^[^ ^37^ ^]^ The MST of 36 days and %ILS of 89.5% were significantly better than other treatment groups (Figure 4C). All considered, the data in Figure 4, strongly support the ability of IRIN to induce an immune response that is augmented by anti‐PD‐1 treatment.

### 2.6 Demonstration of an IRIN‐Induced Immune Response in the Orthotopic KPC Model

**Figure 5:**
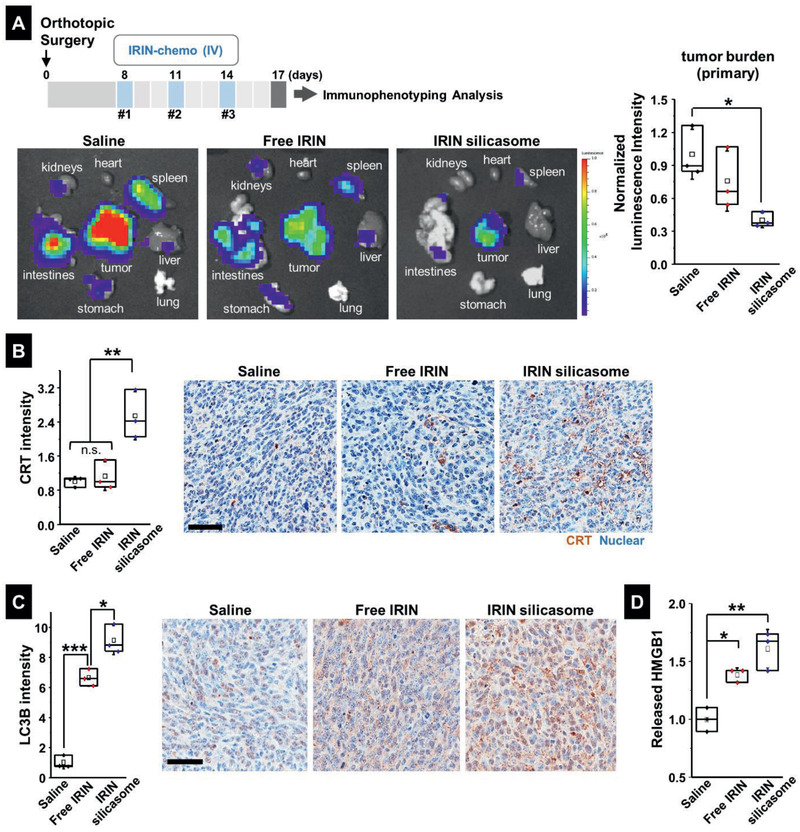
Efficacy study in the KPC model to demonstrate the generation of ICD markers by the IRIN silicasome. A) This experiment was undertaken to demonstrate the immunogenic effect of IRIN in orthotopic tumor‐bearing mice receiving 3 IV injections of either free IRIN or the silicasome at a drug dose of 40 mg kg^−1^, followed by animal sacrifice 72 h after the last treatment. IVIS imaging was performed on explanted organs to obtain the bioluminescence intensity in the region of the primary tumor as well as the metastases. The datawere quantitatively displayed as normalized values by IVIS software in the left panel. IHC analysis to determine B) CRT B) and C) LC3B expression at the orthotopic tumor site. Imaging intensity was quantitatively expressed as fold increase that was normalized to the saline group. Representative IHC images are shown on the right. Bar is 50 µm. D) Quantitative assessment of HMGB1 release. The IHC images were analyzed by Aperio ImageScope software to determine protein released from the damaged nuclei, as described in Figure S15 (Supporting Information). Data are expressed as mean ± SEM, *n* = 3. *p < 0.05; **p < 0.01; ***p < 0.001 (1‐way ANOVA followed by a Tukey's test).

In order to assess whether the innate and cognate arms of the immune system are involved in the response to IRIN, and efficacy experiment, coupled with the assessment of immune response markers, was carried out in animals treated with saline, free IRIN, or the IRIN silicasome (**Figure** **5**A). The orthotopic tumor‐bearing mice (*n* = 3 per group) received IV injection to deliver an IRIN dose equivalent of 40 mg kg^−1^ on days 8, 11, and 14. Animals were sacrificed on day 17 for tumor harvesting to perform ex vivo IVIS imaging as well as IHC analysis for immunogenic responses. From a global tumor growth perspective, quantitative assessment of bioluminescence intensity in “regions of interest” was obtained from the IVIS images, to demonstrate significant tumor shrinkage (*p* < 0.05) in animals treated with the IRIN silicasome compared to saline control (Figure 5A). While free IRIN also reduced tumor growth, the results were not statistically significant (Figure 5A). The IVIS data were further confirmed by tumor weight assessment, as outlined in Figure S14 (Supporting Information).

IHC analysis to assess CRT expression demonstrated that encapsulated IRIN delivery was associated with significantly higher expression of the “eat‐me” biomarker (*p* < 0.01) compared to the staining intensity in animals treated with saline or free drug (Figure 5B). Representative images are shown on the right side (Figure 5B). IHC staining using an antibody that recognizes LC3B also demonstrated significantly increased staining intensity of the autophagy marker in tumor tissue obtained from animals treated with either free or encapsulated IRIN (*p* < 0.001) (Figure 5C). However, staining intensity was significantly higher for encapsulated versus free drug delivery (*p* < 0.05). Representative IHC images appear on the right‐hand side (Figure 5C). Essentially similar results were obtained for HMGB1 staining (Figure 5D), which required the software analysis to be adapted to quantify the amount of released protein from the damaged nuclei, as explained in Figure S15A (Supporting Information). Representative IHC images are shown in Figure S15B (Supporting Information).

**Figure 6:**
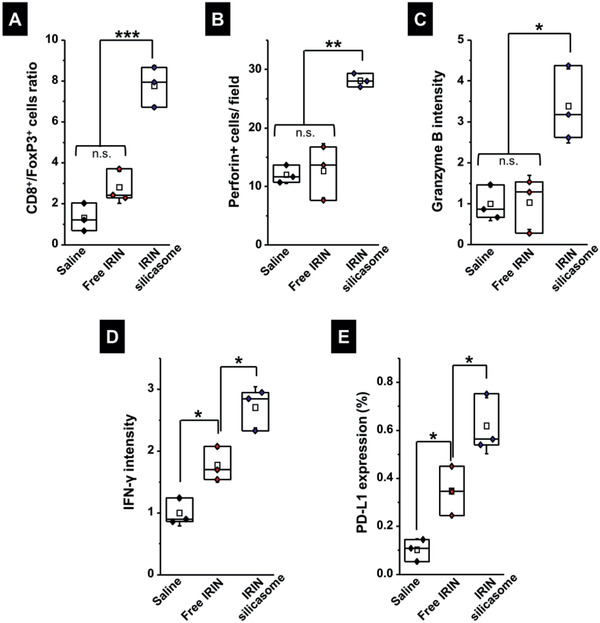
Analysis of cognate immunity in the efficacy experiment in Figure 5. Additional IHC analysis was undertaken using the tumor samples collected in Figure 5A. Quantitative assessment of: A) the CD8^+^/ FoxP3^+^ cell ratio; B) perforin; C) granzyme B; D) IFN‐*γ* production; and E) PD‐L1 expression. Representative IHC images appear in Figure S11A–C (Supporting Information). Data are expressed as mean ± SEM, *n* = 3. *p < 0.05; **p < 0.01; ***p < 0.001 (1‐way ANOVA followed by a Tukey's test).

IHC analysis was also used to assess the expression of CD8^+^ T cells and FoxP3^+^ Treg cells, as shown in the vaccination experiment (Figure 2D). While the silicasome treatment showed a marginal effect on the CD8^+^ T cell number, there was a dramatic reduction of Treg numbers at the tumor site (Figure S16, Supporting Information). This resulted in a significant increase in the CD8^+^/Treg ratio (*p* < 0.001) compared to free drug or the saline control (**Figure** **6**A). We also confirmed increased staining for perforin (*p* < 0.01) and granzyme B (*p* < 0.05) at the tumor site of animals treated with the IRIN silicasome compared to free drug or the saline control (Figure 6B,C). Representative IHC images appear in Figure S17A (Supporting Information). Assessment of IFN‐*γ* production in the TME showed a significant increase in response to treatment by free and encapsulated IRIN, the latter being significantly (*p* < 0.05) higher than free drug (Figure 6D). Representative IHC images appear in Figure S17B (Supporting Information). Similar response profiles were obtained during the assessment of PD‐L1 expression (Figures 6E and Figure S17C, Supporting Information). This is congruent with the level of IFN‐*γ* production, which is a robust inducer of PD‐L1 expression.^[^ ^24^ ^]^

All considered, the IRIN silicasome was more effective than free drug for the ability to induce innate and adaptive anti‐PDAC immune responses at the tumor site. We have previously demonstrated that this is the result of improved pharmacokinetics and drug delivery by the silicasome as a result of its increased carrier stability, circulatory half‐life, and ability to transcytose to the PDAC site.^[^ ^16^ ^]^ Utilizing liquid chromatography (LC)–mass spectrometry (MS), we confirmed that at an IV injection dose of 40 mg kg^−1^,the amount of delivered IRIN in tumor at 24 h is at least a log‐fold higher for encapsulated compared to free drug (Figure S18, Supporting Information). This is consistent with our previous observations in the KPC model.^[^ ^16c^ ^]^ While it is difficult to quantify the amount of free drug in the tumor site, including in cancer cells and the surrounding interstitium, it is reasonable to expect that the acidifying conditions in the PDAC matrix^[^ ^38^ ^]^ assist drug release, as demonstrated in our abiotic study, where ∼20% of the encapsulated irinotecan is released within 4 h at a pH of 4.5 (Figure S19, Supporting Information).

### 2.7 Comparison of the Combination Immunotherapy Response of the IRIN Silicasome versus Onivyde

**Figure 7:**
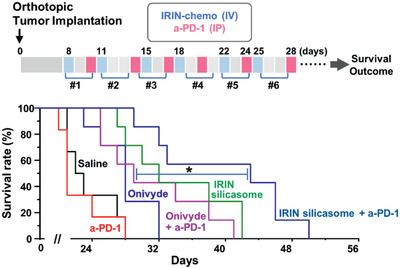
Comparative antitumor immune response for IRIN delivery by the silicasome versus Onivyde, w/wo anti‐PD‐1. The treatment schedule is outlined in the upper panel. Orthotopic KPC tumor‐bearing mice (*n* = 6) were IV injected with Onivyde or the silicasome at an IRIN dose of 40 mg kg^−1^ every 3–4 days, w/w anti‐PD‐1 (100 µg per mouse) IP, two days later. The controls included saline and anti‐PD‐1 antibody alone. Kaplan–Meier plots were used to display the differential survival rate of the different treatment groups (*p < 0.05, Log Rank test).

We also performed a second survival experiment in the KPC orthotopic model to compare the effect of encapsulated IRIN delivery by the silicasome versus the Onivyde liposome^[^ ^3^ ^]^ in the absence and presence of anti‐PD‐1 treatment. The study design, dosimetry considerations, and frequency of administration were the same as in Figure 4B, with minor modifications (**Figure** **7**). While Onivyde improved survival outcome compared to saline, the IRIN silicasome showed additional survival benefit over Onivyde. Moreover, combination therapy with anti‐PD‐1 significantly extended the animal life span during treatment with the IRIN silicasome (comparable to Figure 4B), which was significantly better (*p* < 0.05) than the effect of anti‐PD‐1 coadministration with Onivyde. The response of Onivyde versus Onivyde plus anti‐PD1antibody was nonsignificant (*p* = 0.22). All considered, these data demonstrate that IRIN silicasome plus anti‐PD‐1 combination therapy outperforms Onivyde plus anti‐PD‐1.

## 3 Discussion

In this study, we demonstrate that irinotecan is capable of triggering a chemo‐immunotherapy response in an orthotopic KPC model. Not only is the response more robust during drug delivery by silicasome but considerably augmented in combination with an anti‐PD‐1 antibody. We also show that the immunogenic effects of IRIN, either in free for encapsulated form, can be ascribed to its effect on neutralizing the acidic pH of lysosomes. This early effect leads to autophagy inhibition and the triggering of an ER stress response that culminates in a “Type II” ICD response and PD‐L1 overexpression. These immunogenic effects were confirmed by the ability of exposed KPC cells to trigger a vaccination response in vivo. The ability to induce an anti‐tumor immune response was further confirmed in an orthotopic KPC tumor model, in which the delivery of IRIN to the tumor site by a silicasome could be seen to induce ICD markers, in addition to increasing the CD8/Treg ratio and PD‐L1 expression. Moreover, response augmentation by anti‐PD‐1 antibodies was seen to significantly prolong animal survival, far superior to the use of anti‐PD‐1 in combination with free drug or Onivyde. These results suggest that it is feasible to improve PDAC survival with the IRIN silicasome through a chemo‐immunotherapy effect that generates a “hot” tumor microenvironment that can be further exploited by immune checkpoint therapy.

IRIN has been used to treat solid tumors for ≈30 years, particularly in patients with PDAC, colorectal and certain types of lung cancer.^[^ ^39^ ^]^ Similar to the most cancer drug, it is a potent cancer drug with pleiotropic cellular effects. The classic mode of action (MOA) of this cytotoxic alkaloid is its conversion to SN‐38, which functions as a topoisomerase I inhibitor, capable of inducing single and double strand DNA breaks.^[^ ^40^ ^]^ It is not a surprise, therefore, that most studies addressing IRIN anticancer effects have focused on damage to the cell nucleus, without paying much attention to extranuclear effects.^[^ ^39^, ^40^ ^]^ However, studies of the impact of IRIN on colon cancer cell death have revealed evidence of “lysosomal leakage” and a possible impact on autophagy.^[^ ^41^ ^]^ We now characterize this as an impact on autophagy flux due to the weak basic properties of IRIN. This leads to alkalization of the lysosomal pH by the free as well as the encapsulated drug. These IRIN medicinal chemistry features (pKa=8.1; LogP=2.78) are characteristic of lysosomotropic agents that exhibit pKa values of 7.5‐10.5 and LogP values of 2.5‐5.5,^[^ ^42^ ^]^ therefore, it is reasonable to expect that once IRIN reaches lysosome, the 1^st^ mode of action of this weak‐base is proton consumption, similar to other lysosomotropic agents such as acridine orange^[^ ^43^ ^]^ and chloroquine.^[^ ^44^ ^]^ The latter was included as a positive control in Figure 1E. Nevertheless, the lysosomal alkalization effect results in interference in organelle fusion with autophagosomes (Figures 1 and 3). Subsequent accumulation of p62, results in increased expression of PD‐L1 (Figures 1 and 3). This distinguishes IRIN from most classic ICD inducers that are capable of inducing rather than interfering in autophagy.^[^ ^12^, ^14^ ^]^ While the exact mechanism by which p62 induces PD‐L1 expression requires further study, some evidence has been obtained that this could involve NF‐*κ*B activation.^[^ ^23^ ^]^ This is in keeping with our data showing increased phosphorylation of the p65 NF‐*κ*B subunit (Figure S4, Supporting Information). In addition to activating a potential immune escape mechanism, IRIN is also capable of inducing a “Type II” ICD, primarily linked to ER stress (Figure 2). This is important since this drug until now has been depicted as having an ICD status that is “nondetermined.”^[^ ^12e^ ^]^ We do show, however, that IRIN is capable of inducing CRT expression and HMGB1 release, in addition to the ability to generate a robust vaccination and life‐prolonging immune response in the orthotopic PDAC model (Figure 5B–D). This is equivalent to an endogenous vaccination response that holds the promise to switch the "cold" immunogenic PDAC microenvironment into "hot" status with activated cytotoxic T‐cells. This may improve the responsiveness to anti‐PD‐1 (Figure 4). All considered, our study provides cumulative evidence that IRIN can indeed play an immunogenic role in cancer, as outlined in **Table** **1**.^[^ ^45^ ^]^ Further evidence for the involvement of the immune system is the augmentation of MHC class I expression, concurrent with increased PD‐L1 expression on mammary tumor cells.^[^ ^45a^ ^]^ Moreover, in a mammary carcinoma model, it has also been demonstrated that IRIN can induce Treg depletion, in addition to the potential to synergize with anti‐PD‐L1.^[^ ^45a^ ^]^ Further, a subcutaneous MC38/gp100 colon cancer model was used to demonstrate that the antitumor efficacy of an IRIN‐delivering liposome can be enhanced by ICI antibodies.^[^ ^45b^ ^]^

**Table 1:** Comparison of IRIN chemo plus anti‐PD‐1/anti‐PD‐L1 combination in the literature and this study

Ref.	Treatments	Cancer model	Anticancer effect	Key immunological and other biological findings of IRIN
^[^ ^45a^ ^]^	Free IRIN w/wo a‐PD‐L1	Subcutaneous FM3A Breast Cancer	Potency ranking: Free IRIN + a‐PD‐L1 > Free IRIN ≈ a‐PD‐L1 > saline	CD8/Treg ratio ↑ MHC class I ↑ PD‐L1 ↑
^[^ ^45b^ ^]^	Liposomal IRIN (Onivyde/MM398) w/wo a‐PD‐1/a‐PD‐L1	SubcutaneousMC38/gp100 Colon Cancer	Potency ranking: Liposomal IRIN + a‐PD‐1/ a‐PD‐L1 > liposomal IRIN a‐PD‐1/ a‐PD‐L1 alone ≈ saline	CD8/Treg ratio ↑ Granzyme B^+^ T cells ↑
This study	Free IRIN w/wo a‐PD‐1 ONIVYDE w/wo a‐PD‐1 IRIN silicasome w/wo a‐PD‐1	Orthotopic KPC Pancreatic Cancer	Potency ranking: IRIN silicasome + a‐PD‐1 > IRIN silicasome ≈ IRIN + a‐PD‐1 > free IRIN ≈ a‐PD‐1 ≈ saline IRIN silicasome + a‐PD‐1 > IRIN silicasome ≈ ONIVYDE + a‐PD‐1 > ONIVYDE > a‐PD‐1 ≈ saline [Note: “IRIN silicasome + a‐PD‐1” exhibited the longest survival outcome]	CD8/Treg ratio ↑ PD‐L1 expression ↑ Granzyme B^+^ T cells ↑ Perforin^+^ T cells ↑ IFN‐*γ* production ↑ ICD (CRT, HMGB1) and ER stress ↑ Enrichment in acidic vesicles and autophagy inhibition ↑

Since its approval in 1998, the development of new IRIN formulations has been an area of great interest because of this drug's high potency, which also leads to severe dose‐limiting side effects.^[^ ^39^ ^]^ This includes severe neutropenia and gastrointestinal toxicity, which limits IRIN use to PDAC patients with good clinical status. IRIN also has the shortcoming of large interindividual PK variability that is accentuated by poor access to the desmoplastic PDAC tumor site.^[^ ^34^ ^]^ These challenges provided the basis for developing the liposomal formulation that received FDA approval as Onivyde.^[^ ^3^ ^]^ While successful for inducing PDAC responses in the clinic, Onivyde received a black box warning for residual drug toxicity, which could be due to drug leakage by the unsupported lipid bilayer.^[^ ^3^ ^]^ This observation promoted the development of the IRIN‐silicasome, which makes use of a supported lipid bilayer.^[^ ^16a,c^ ^]^ From this perspective, the silicasome can be viewed as a next‐generation liposome (Figure 3A), which is less leaky, and capable of reducing bone marrow and gastro‐intestinal toxicity compared to Onivyde^[^ ^3^ ^]^ or an in‐house liposome in orthotopic KPC as well as colon cancer models.^[^ ^16a,c^ ^]^ Moreover, the increased stability of the silicasome also contributes to improved drug delivery at the desmoplastic PDAC tumor site.^[^ ^16^, ^34^ ^]^ In light of our demonstration of the unusual immunogenic properties of IRIN, we introduce a novel treatment option for the use of the silicasome for PDAC treatment in the clinic in combination with ICI antibodies. We are also aware of an ongoing clinical trial that is looking at triple therapy (Onivyde/5‐fluorouracil/leucovorin) with anti‐PD‐1 antibody (pembrolizumab) plus a CXCR4 antagonist (BL‐8040) for metastatic PDAC in patients who failed gemcitabine therapy (NCT02826486).^[^ ^46^ ^]^ While the final report is not released yet, the preliminary data are encouraging, showing an objective response rate (ORR) of 32% for triple‐therapy versus 17% for chemo combination only.^[^ ^3^, ^46^ ^]^

While most ICD responses are categorized as “Type I” responses, “Type II” responses have been linked to agents that generate a primary ER stress response and ROS production, e.g., hypericin‐induced photodynamic therapy^[^ ^47^ ^]^ and Pt‐N‐heterocyclic carbene.^[^ ^48^ ^]^ We now demonstrate that IRIN, both as a free or an encapsulated drug format, induces robust ROS production and ER stress, and CHOP expression (Figures 1, 2, 3). Moreover, its lysosomal effects appear to precede the onset of nuclear damage, suggesting that the ER stress effect is a primary response. This particular constellation of findings would place IRIN into the category of “Type II” ICD inducer, which sets it apart from other chemotherapeutic agents such as OX. This could be advantageous from the perspective that Type II inducers are considered more robust inducers of anti‐tumor immune responses.^[^ ^14^, ^26^ ^]^ A more robust ICD effect holds clear advantages from the perspective that PDAC is generally considered a poorly immunogenic tumor.^[^ ^5^, ^49^ ^]^ Not only does this open up the possibility for combination therapy with ICIs, as we show in the use of anti‐PD‐1 (Figures 4 and 7), but it also paves the way for considering the introduction of additional immunomodulators, including anti‐CD40 antibodies, IDO‐1 inhibitors, autophagic inhibitors, or small drug inhibitors of immune checkpoint pathways.^[^ ^50^ ^]^ From this perspective, the creative use of the multifunctional silicasome platform promises to allow additional synergistic treatment combination for use in immunotherapy. Here it is important to consider the contribution of the dysplastic stroma, its high content of carcinoma‐associated fibroblasts (CAFs) and myeloid derived dendritic cells to interference in the PDAC immune response.^[^ ^5^, ^10^, ^49^ ^]^ This can be accomplished by combinatorial therapy with TGF‐*β* inhibitor (e.g., LY364947),^[^ ^15^, ^51^ ^]^ as well as CXCR4 antagonists (e.g., AMD 3100^[^ ^52^ ^]^ and BL‐8040^[^ ^46^ ^]^), which can disrupt adhesive tumor‐stroma interactions and overcome T cell exclusion mechanism via targeting FAP‐expressing CAFs, making PDAC more accessible to conventional drugs and cytotoxic T cells.^[^ ^46^, ^52^, ^53^ ^]^

## 4 Conclusions

In summary, we provide a novel explanation for the immunogenic effects of irinotecan. First, the weak basic drug neutralizes the acidic pH of the lysosome in KPC cells, leading to autophagy inhibition and upregulation of PD‐L1 expression. Another linked effect is the delivery of a robust ER stress response which leads to cell death, characterized by ecto‐CRT expression and the generation of immunological danger signals. Collectively, this culminates in an immunogenic cell death response accompanied by PD‐L1 expression. The in vivo relevance is that this allowed us to induce an ICD response in an orthotopic KPC model by using encapsulated delivery of IRIN in a silicasome carrier. The response could be augmented by anti‐PD‐1 treatment, leading to a pronounced survival improvement compared to anti‐PD‐1 combination therapy with free drug or a liposome composition. Our discovery introduces a major additional avenue for PDAC chemotherapy.

## 5 Experimental Section

##### Materials

Tetraethylorthosilicate (TEOS), triethanolamine (TEA‐ol), triethylamine (TEA) cetyltrimethylammonium chloride solution (CTAC, 25 wt% in water), Dowex 50WX8 resin, and chloroquine diphosphate salt were purchased from Sigma‐Aldrich, USA. Sucrose octasulfate (SOS) sodium salt was purchased from Toronto Research Chemicals, Inc, Canada. 1,2‐Distearoyl‐*sn*‐glycero‐3‐phosphocholine (DSPC), 1,2‐distearoyl‐*sn*‐glycero‐3‐phospho‐ethanol amine‐*N*‐[methoxy(polyethylene glycol)‐2000] (ammonium salt) (DSPE‐PEG_2000_), and cholesterol (Chol) were purchased from Avanti Polar Lipids, USA. Sepharose CL‐4B was purchased from GE Healthcare, USA. Irinotecan hydrochloride trihydrate, oxaliplatin, doxorubicin hydrochloride salt, paclitaxel, and rapamycin were purchased from LC Laboratories, USA. Tunicamycin was purchased from Cell Signaling Technology. Onivyde (Ipsen Biopharmaceuticals, Inc., 4.3 mg mL^−1^ irinotecan free base, 10 mL per vial) was purchased through the UCLA Health Pharmacy. Murine anti‐PD‐1 antibody (#BE0146) and dilution buffer (#IP0070) in InVivoPure were purchased Bio X Cell. Penicillin, streptomycin, Dulbecco's modified Eagle medium (DMEM) and LysoTracker Red DND‐99 (L7528), and Fluo‐4 AM (F14201) were purchased from Invitrogen. Cellular ROS Assay Kit (Red) (ab186027) was purchased from Abcam. Fetal bovine serum (FBS) was purchased from Gemini Bio Products. Murine IFN‐*γ* was purchased from R&D (Minneapolis, MN). Matrigel Matrix Basement Membrane was purchased from BD Bioscience.

##### Cell Culture

The KPC pancreatic adenocarcinoma cell line, which was derived from a spontaneous tumor originating in a transgenic Kras^LSL‐G12D/+^; Trp53^LSL‐R172H/+^; Pdx‐1‐Cre mouse (B6/129 background),^[^ ^16^, ^17^ ^]^ was cultured in DMEM, containing 10% FBS, 100 U mL^−1^ penicillin, 100 µg mL^−1^ streptomycin, 2 × 10^−3^ m l‐glutamine and 1 × 10^−3^ m sodium pyruvate. To allow bioluminescence tumor imaging, the cells were permanently transfected with a luciferase‐based lentiviral vector in the UCLA vector core facility, followed by a limiting dilution cloning as previously described.^[^ ^16a^ ^]^ PANC‐1 cells were obtained from the American Type Culture Collection (ATCC), and cultured under similar conditions as KPC cells.

##### Use of Confocal Microscopy to Study the Intracellular Distribution and Alkalinizing Effect of IRIN‐treated KPC Cells

Approximately 1.5 × 10^4^ KPC cells were seeded in µ‐Slide 8 well (ibidi, 80826). To reveal the intracellular distribution of IRIN, attached KPC cells were treated with IRIN (300 × 10^−6^ m) for 24 h. Cell membranes were stained using an Alexa Fluor 594 conjugated WGA dye (Invitrogen, W11262) at 2 µg mL^−1^ for 10 min. After washing, the cells were visualized using a Leica SP8‐MD confocal microscope under the 100× objective lens. The excitation and emission wavelengths for IRIN were 405 and 440–490 nm, respectively.^[^ ^54^ ^]^

To demonstrate the alkalinizing cellular effect of IRIN, LysoTracker Red DND‐99 was used. IRIN‐treated KPC cells were washed and replenished with fresh media containing 100 × 10^−9^ m DND‐99 dye and 5 µg mL^−1^ Hoechst 33342 for nuclear staining. The cells were incubated at 37 °C for 0.5 h, followed by washing with phenol red free media. The cells were then visualized using a Leica SP8‐MD confocal microscope under the 100× objective lens.

##### Use of IF Staining to Demonstrate the Effect of IRIN on Autophagy and PD‐L1 Expression in KPC Cells

KPC cells were seeded in the µ‐Slide 8 well. After cell attachment, the cells were treated by IRIN at 300 × 10^−6^ m for 24 h. Control treatments included exposure to PBS, chloroquine (32 × 10^−6^ m), rapamycin (100 × 10^−9^ m), and IFN‐*γ* (10 ng mL^−1^). Before IF staining, the cells were washed with PBS and fixed with 4% paraformaldehyde at RT for 15 min. Cells were treated in 1% BSA (blocking reagent) plus 0.2% Triton‐X100 in PBS for 30 min. Subsequently, the cells were incubated with primary antibody that recognizes LC3B (Cell Signaling #2775, 1:200) or p62/SQSTM1 (Cell Signaling #23214, 1:800) in 1% BSA containing PBS solution at 4 °C overnight. The sample was washed twice in PBS and further stained using an Alexa Fluor 488 conjugated goat anti‐rabbit secondary antibody (Thermo Fisher, A‐11008, 1:1000) and 5 µg mL^−1^ Hoechst 33342 dye for 1 h. Cell surface staining for PD‐L1 assessment was carried out in fixed (4% paraformaldehyde) cells, using a staining process similar to the above protocol. Primary PD‐L1 antibody (Abcam, ab213480, 1:500) and Alexa Fluor 594 conjugated goat antirabbit secondary antibody (Thermo Fisher, A‐11012, 1:1000) were used. The cells were visualized using a Leica SP8‐MD confocal microscope under the 100× objective lens.

##### Western Blotting

To confirm the confocal staining results for LC3B, p62 and PD‐L1, western blotting experiments were performed in KPC cells exposed to IRIN (300 × 10^−6^ m) or OX (500 × 10^−6^ m) for 24 h. Another experiment also looked at the dose‐dependent effect of IRIN at concentrations of 75, 150, 300, and 600 × 10^−6^ m for 24 h. Briefly, ≈2 × 10^5^ KPC cells per well were seeded in 6‐well plates. After drug exposure, KPC cells were harvested and treated with cold RIPA lysis buffer (Cell Signaling # 9806S), supplemented with a cocktail of protease and phosphatase inhibitors (Cell Signaling #5872) and incubated on ice for 30 min. After centrifugation of the lysates at 12 000 rpm for 10 min, protein concentration was quantified by a Bradford assay (Biorad). Equal amounts of protein in the supernatants were loaded onto a 10–20% Tris‐glycine SDS‐PAGE gel (Invitrogen, Grand Island, NY). The proteins were subsequently transferred to a PVDF membrane. The membrane was blocked with 5% nonfat dry milk/TBST, before incubation with primary and HRP‐conjugated secondary antibodies. The primary antibodies included: LC3B (Cell Signaling #2775), p62/SQSTM1 (Cell Signaling #5114), NF‐*κ*B p65 (Cell Signaling #8242), Phospho‐NF‐*κ*B p65 (Cell Signaling #3033), and PD‐L1 (Abcam, ab213480). The blots were developed by soaking in ECL substrate (Thermo Fisher Scientific). Densitometric analysis of each protein band on the film was quantified by ImageJ software and normalized to the intensity of a corresponding housekeeping protein.

Western blotting was also used to detect the ER stress marker, CHOP, and Cleaved Caspase‐3 (CC‐3). In this case, KPC cells were treated with IRIN at the indicated concentrations (75 × 10^−6^–600 × 10^−6^ m) for 24 h. In a separate experiment, KPC cells were treated with IRIN (300 × 10^−6^ m) and tunicamycin (10 × 10^−6^) for 4 h. Primary antibodies were purchased from Cell Signaling Technology: *β*‐actin (#3700), vinculin (#13901), CHOP (#2895), and Cleaved Caspase‐3 (#9664).

##### Measurement of the IRIN Effect on CRT Expression and HMGB1 Release

7.5 × 10^4^ KPC cells were seeded into 24‐well plates. After cell attachment, KPC cells were treated with IRIN (300 × 10^−6^ m), OX (500 × 10^−6^ m), DOX (20 × 10^−6^ m), or PTX (12 × 10^−6^ m) for 24 h. The cell culture media were collected in 1.5 mL tube and spun down (2000 rpm for 5 min) to collect the supernatants for HMGB1 detection by an ELISA kit (Catalog# ST51011, IBL International GmbH). Surface CRT expression was measured by flow cytometry in the same experiment, as previously described.^[^ ^27^ ^]^ Briefly, the loosely attached cells were combined with trypsin‐treated adhered cells. The cells were washed in cold PBS and then stained with a primary anti‐CRT antibody (Abcam, ab2907, 1:140) in 200 µL BD staining buffer for 0.5 h on ice. The cells were washed in cold PBS and stained with an Alexa Fluor 680‐conjugated secondary antibody (LifeScience Technologies #A21244) for 30 min on ice. After washing in cold PBS, the cells were assessed in a LSRII flow cytometer (BD Biosciences).

##### Animal Purchase and Study Approval

Female B6/129SF1/J mice (JAX 101043) were purchased from The Jackson Laboratory, and maintained under pathogen‐free conditions. All animal experiments were performed according to protocols approved by the UCLA Animal Research Committee.

##### Assessment of the Immunogenic Effects of IRIN in a Vaccination Experiment

The vaccination schedule is highlighted in Figure 2C. Eight million KPC cells were seeded in a tissue culture dish. After cellular attachment, IRIN (300 × 10^−6^ m) or OX (500 × 10^−6^ m) were added for 24 h. Cells were collected and washed before resuspended in 0.8 mL cold PBS. For vaccination, each mouse received subcutaneous (SC) injection of a 100 µL suspension of chemo‐treated cells in the right flank. Control animals received SC injection with 0.1 mL PBS. The vaccination was repeated after 7 days. Fourteen days after the 1st injection, the animals received SC injection of normal KPC cells (1 million cells in 0.1 mL PBS) in the contralateral (left) flank. Tumor growth was measured by a digital caliper every 2–3 days, and the tumor volume calculated according to the formula: length × width^2^ /2. Animals were sacrificed on day 26 and the tumors were collected, weighed and fixed in 10% formalin, followed by paraffin embedding and sectioning to derive to 4 µm thick slices for IHC analysis. Primary antibodies to CD8 (#14‐0808‐82) and FoxP3 (#13‐5773‐82) were purchased from ThermoFisher. IHC staining was performed in the UCLA Translational Pathology Core Laboratory (TPCL). The slides were scanned and images assessed by using Aperio ImageScope software (Leica).

##### Synthesis, Purification, and Characterization of IRIN Silicasomes

The irinotecan‐loaded silicasomes were prepared as previously reported described.^[^ ^16c^ ^]^ Briefly, bare MSNPs were synthesized at 18 L scale and purified by extensive acidic ethanol washing to remove the CTAC detergent.^[^ ^16c^ ^]^ The trapping agent (TEA_8_SOS) was prepared from a sucrose octasulfate sodium salt. For lipid coating, 40 mg mL^−1^ of the purified MSNPs, exposed to 80 × 10^−3^ m TEA_8_SOS solution for soaking in, were added to an ≈50% (w/v) lipid solution. This ethanol suspended solution contained DSPC/Chol/DSPE‐PEG_2000_, in the molar ratio of 3:2:0.15. This yields a MSNP:lipid ratio of 1:1.25 (w/w). The suspension was introduced by a flow pump into a flow cell (Sonics & Materials, Inc., #53630‐0651) that provides probe sonication (Ultrasonic Processor Model VCX500, 80% amplitude) at a 15 s/15 s on/off cycle and a flow rate of 5 mL min^−1^.^[^ ^16c^ ^]^ In order to remove the free trapping agent, the sample was purified through centrifugation (4000 rpm for 5 min), followed by purification, using size exclusion chromatography. For IRIN import, TEA_8_SOS‐loaded particles were mixed with IRIN and incubated at 65 °C for 1 h. The IRIN silicasomes were purified and filtered across a 0.2 µm filter for sterilization.

The final product was fully characterized as previously described by us.^[^ ^16c^ ^]^ Briefly, the loading capacity was calculated as the weight ratio of irinotecan to MSNP. MSNP mass was determined by TGA. Particle hydrodynamic size and zeta potential were measured by a ZETAPALS instrument (Brookhaven Instruments Corporation). The final product was visualized by cryoEM (TF20 FEI Tecnai‐G2) to confirm the uniformity and integrity of the coated lipid bilayer. A chromogenic LAL assay (QCL‐1000 300 Test Kit, Lonza) was performed to test the endotoxin levels. Sterilization of the final product was confirmed by microbial (HPC Count sampler, Millipore Corp., MHPC10025), yeast and mold counting (Yeast and mold sampler, Millipore Corp., MY0010025) tests.

##### Assessment of Cellular Responses to the IRIN Silicasome

The IRIN silicasome on lysosomal alkalization was performed at drug concentrations of 75 × 10^−6^ and 300 × 10^−6^ m in KPC cells. Empty silicasomes (in which a 500 µg mL^−1^ particle dose is representative of an encapsulated IRIN dose of 300 × 10^−6^ m) was included as control. The assessment of LC3B, p62, and PD‐L1 immunoblotting and IF staining were performed in KPC cells during treatment with the IRIN silicasome or empty silicasomes, as described for the free drug.

##### Assessment of the Treatment Response to Combination Therapy with the IRIN Silicasome Plus Anti‐PD‐1 in an Orthotopic KPC Tumor Model

The KPC‐derived orthotopic tumor model in immunocompetent B6129SF1/J mouse was established as described in Figure 4A.^[^ ^16^ ^]^ Briefly, 30 µL of DMEM/Matrigel (1:1 v/v), containing ≈1 × 10^6^ KPC‐luc cells, was injected into the tail of the pancreas in female B6129SF1/J mice (8–10 weeks) by a short survival surgery procedure.^[^ ^16^ ^]^ In the first survival experiment (Figure 4B), tumor‐bearing mice were randomly assigned into 6 groups (*n* = 5–7) and received IV injection of the IRIN formulations at API dose of 40 mg kg^−1^. Anti‐PD‐1 antibody was injected at 100 µg per animal IP. To assess survival rate, animals were monitored daily up to the stage of spontaneous death or approaching moribund status (defined in Figure 4A).^[^ ^16^, ^36^ ^]^ The survival data were plotted as Kaplan–Meier curves, followed by data analysis to derive mean survival time. Statistical analysis and *p* values were obtained by Log Rank testing (Mantel‐Cox), using GraphPad Prism 7.00 software.

##### Assessment of Immune Parameters in Response to Silicasome Treatment in the Orthotopic KPC Tumor Model

Tumor‐bearing mice received IV injection to deliver an IRIN dose of 40 mg kg^−1^ (free IRIN or IRIN silicasome) per injection every 3 days for a total of 3 administrations. Control animals received saline only. Animals were sacrificed 72 h after the last injection. To confirm the impact on tumor growth, ex vivo bioluminescence imaging was performed to assess image intensity at the primary and metastatic tumor sites. Primary tumors were fixed in 10% formalin, followed by paraffin embedding and sectioning to provide 4 µm slices for IHC analysis in the UCLA Translational Pathology Core Laboratory (TPCL). The slides were scanned and images were assessed by using Aperio ImageScope software (Leica). Primary antibodies to CD8 (#14‐0808‐82) and FoxP3 (#13‐5773‐82) were purchased from ThermoFisher; CRT (ab2907), while antibodies to HMGB1 (ab18256), granzyme B (ab4059), perforin (ab16074) and IFN‐*γ* (ab9657) were purchased from Abcam. The antibody to LC‐3 (#0231‐100/LC3‐5F10) was purchased from Nanotools, while the antibody to PD‐L1 (#64988) was purchased from Cell Signaling Technology.

##### Assessment of Anti‐PD1 Combination Therapy with the Silicasomes versus Onivyde in the Orthotopic KPC Model

The treatment schedule and frequency of administration are outlined in Figure 7. Orthotopic KPC tumor‐bearing mice were randomly assigned into 6 groups (*n* = 6). The treatment groups included: animals receiving IV injection of IRIN (Onivyde or IRIN silicasome) at 40 mg kg^−1^; IP injections of anti‐PD‐1 antibody monotherapy (100 µg per injection); or an anti‐PD‐1/chemo combination, for a total of 6 administrations. Kaplan–Meier analysis was performed as described in Figure 6.

##### Statistical Analysis

Comparative analysis of differences between groups was performed using the 2‐tailed Student's *t*‐test (Excel software, Microsoft) for two‐group comparison. One‐way ANOVA followed by a Tukey's test (Origin software, OriginLab) was performed for multiple group comparisons. Data were expressed as mean ± SD or scanning electron microscopy (SEM), as stated in the figure legends. The survival analysis was performed by Log Rank testing (Mantel‐Cox), using GraphPad Prism 7.00 software. A statistically significant difference was considered at *p < 0.05.

## Conflict of Interest

A.E.N. and H.M. are co‐founders, board members, and equity holders in Westwood Bioscience Inc. UCLA entered into a sponsored research agreement with Westwood Bioscience Inc. A.E.N. and H.M. are co‐founders and equity holders in NAMMI therapeutics. The remaining authors declared no conflict of interest.

## Supporting information

Supporting Information

## References

[1] a) F. Bray , J. Ferlay , I. Soerjomataram , R. L. Siegel , L. A. Torre , A. Jemal , CA‐Cancer J. Clin. 2018, 68, 394; 30207593 10.3322/caac.21492

[2] a) F. Yang , C. Jin , D. L. Fu , A. L. Warshaw , World J. Gastroenterol. 2019, 25, 2839; 31249443 10.3748/wjg.v25.i23.2839 PMC6589737

[3] A. Wang‐Gillam , C. P. Li , G. Bodoky , A. Dean , Y. S. Shan , G. Jameson , T. Macarulla , K. H. Lee , D. Cunningham , J. F. Blanc , R. A. Hubner , C. F. Chiu , G. Schwartsmann , J. T. Siveke , F. Braiteh , V. Moyo , B. Belanger , N. Dhindsa , E. Bayever , D. D. Von Hoff , L. T. Chen , N.‐S. Group , Lancet 2016, 387, 545.26615328

[4] a) S. L. Topalian , F. S. Hodi , J. R. Brahmer , S. N. Gettinger , D. C. Smith , D. F. McDermott , J. D. Powderly , R. D. Carvajal , J. A. Sosman , M. B. Atkins , N. Engl. J. Med. 2012, 366, 2443; 22658127 10.1056/NEJMoa1200690 PMC3544539

[5] a) D. Kabacaoglu , K. J. Ciecielski , D. A. Ruess , H. Algul , Front. Immunol. 2018, 9, 1878; 30158932 10.3389/fimmu.2018.01878 PMC6104627

[6] Z. I. Hu , J. Shia , Z. K. Stadler , A. M. Varghese , M. Capanu , E. Salo‐Mullen , M. A. Lowery , L. A. Diaz, Jr. , D. Mandelker , K. H. Yu , A. Zervoudakis , D. P. Kelsen , C. A. Iacobuzio‐Donahue , D. S. Klimstra , L. B. Saltz , I. H. Sahin , E. M. O'Reilly , Clin. Cancer Res. 2018, 24, 1326. 29367431 10.1158/1078-0432.CCR-17-3099 PMC5856632

[7] a) K. C. Soares , A. A. Rucki , A. A. Wu , K. Olino , Q. Xiao , Y. Chai , A. Wamwea , E. Bigelow , E. Lutz , L. D. Liu , S. Yao , R. A. Anders , D. Laheru , C. L. Wolfgang , B. H. Edil , R. D. Schulick , E. M. Jaffee , L. Zheng , J. Immunother. 2015, 38, 1; 25415283 10.1097/CJI.0000000000000062 PMC4258151

[8] a) C. Lu , A. V. Paschall , H. Shi , N. Savage , J. L. Waller , M. E. Sabbatini , N. H. Oberlies , C. Pearce , K. Liu , J. Natl. Cancer Inst. 2017, 109, djw283; 10.1093/jnci/djw283 PMC5291187 28131992

[9] L. Zheng , J. Natl. Cancer Inst. 2017, 109, djw304.

[10] a) A. Henriksen , A. Dyhl‐Polk , I. Chen , D. Nielsen , Cancer Treat. Rev. 2019, 78, 17; 31325788 10.1016/j.ctrv.2019.06.005

[11] a) K. M. Heinhuis , W. Ros , M. Kok , N. Steeghs , J. H. Beijnen , J. H. M. Schellens , Ann. Oncol. 2019, 30, 219; 30608567 10.1093/annonc/mdy551

[12] a) A. D. Garg , D. Nowis , J. Golab , P. Vandenabeele , D. V. Krysko , P. Agostinis , Biochim. Biophys. Acta 2010, 1805, 53; 19720113 10.1016/j.bbcan.2009.08.003

[13] O. Kepp , L. Menger , E. Vacchelli , C. Locher , S. Adjemian , T. Yamazaki , I. Martins , A. Q. Sukkurwala , M. Michaud , L. Senovilla , L. Galluzzi , G. Kroemer , L. Zitvogel , Cytokine Growth Factor Rev. 2013, 24, 311. 23787159 10.1016/j.cytogfr.2013.05.001

[14] a) M. Michaud , I. Martins , A. Q. Sukkurwala , S. Adjemian , Y. Ma , P. Pellegatti , S. Shen , O. Kepp , M. Scoazec , G. Mignot , S. Rello‐Varona , M. Tailler , L. Menger , E. Vacchelli , L. Galluzzi , F. Ghiringhelli , F. di Virgilio , L. Zitvogel , G. Kroemer , Science 2011, 334, 1573; 22174255 10.1126/science.1208347

[15] a) H. Han , D. Valdeperez , Q. Jin , B. Yang , Z. Li , Y. Wu , B. Pelaz , W. J. Parak , J. Ji , ACS Nano 2017, 11, 1281; 28071891 10.1021/acsnano.6b05541

[16] a) X. S. Liu , A. Situ , Y. A. Kang , K. R. Villabroza , Y. P. Liao , C. H. Chang , T. Donahue , A. E. Nel , H. Meng , ACS Nano 2016, 10, 2702; 26835979 10.1021/acsnano.5b07781 PMC4851343

[17] a) S. R. Hingorani , L. Wang , A. S. Multani , C. Combs , T. B. Deramaudt , R. H. Hruban , A. K. Rustgi , S. Chang , D. A. Tuveson , Cancer Cell 2005, 7, 469; 15894267 10.1016/j.ccr.2005.04.023

[18] H. Y. Wei , J. Song , H. Li , Y. Li , S. S. Zhu , X. D. Zhou , X. W. Zhang , L. Yang , Asian J. Pharm. Sci. 2013, 8, 303.

[19] G. Griffiths , B. Hoflack , K. Simons , I. Mellman , S. Kornfeld , Cell 1988, 52, 329. 2964276 10.1016/s0092-8674(88)80026-6

[20] a) A. Mahli , M. Saugspier , A. Koch , J. Sommer , P. Dietrich , S. Lee , R. Thasler , J. Schulze‐Luehrmann , A. Luehrmann , W. E. Thasler , M. Muller , A. Bosserhoff , C. Hellerbrand , Gut 2018, 67, 746; 28053052 10.1136/gutjnl-2016-312485

[21] a) G. Kroemer , M. Jaattela , Nat. Rev. Cancer 2005, 5, 886; 16239905 10.1038/nrc1738

[22] C. Mauvezin , P. Nagy , G. Juhász , T. P. Neufeld , Nat. Commun. 2015, 6, 7007. 25959678 10.1038/ncomms8007 PMC4428688

[23] X. Wang , W. K. K. Wu , J. Gao , Z. Li , B. Dong , X. Lin , Y. Li , Y. Li , J. Gong , C. Qi , Z. Peng , J. Yu , L. Shen , J. Exp. Clin. Cancer Res. 2019, 38, 140. 30925913 10.1186/s13046-019-1148-5 PMC6440013

[24] A. Garcia‐Diaz , D. S. Shin , B. H. Moreno , J. Saco , H. Escuin‐Ordinas , G. A. Rodriguez , J. M. Zaretsky , L. Sun , W. Hugo , X. Wang , G. Parisi , C. P. Saus , D. Y. Torrejon , T. G. Graeber , B. Comin‐Anduix , S. Hu‐Lieskovan , R. Damoiseaux , R. S. Lo , A. Ribas , Cell Rep. 2017, 19, 1189. 28494868 10.1016/j.celrep.2017.04.031 PMC6420824

[25] a) A. D. Garg , A. M. Dudek , G. B. Ferreira , T. Verfaillie , P. Vandenabeele , D. V. Krysko , C. Mathieu , P. Agostinis , Autophagy 2013, 9, 1292; 23800749 10.4161/auto.25399

[26] a) A. D. Garg , A. M. Dudek‐Peric , E. Romano , P. Agostinis , Int. J. Dev. Biol. 2015, 59, 131; 26374534 10.1387/ijdb.150061pa

[27] M. Obeid , A. Tesniere , F. Ghiringhelli , G. M. Fimia , L. Apetoh , J. L. Perfettini , M. Castedo , G. Mignot , T. Panaretakis , N. Casares , D. Metivier , N. Larochette , P. van Endert , F. Ciccosanti , M. Piacentini , L. Zitvogel , G. Kroemer , Nat. Med. 2007, 13, 54. 17187072 10.1038/nm1523

[28] a) C. M. Oslowski , F. Urano , Methods Enzymol. 2011, 490, 71; 21266244 10.1016/B978-0-12-385114-7.00004-0 PMC3701721

[29] H. Yang , H. Shen , J. Li , L. W. Guo , Autophagy 2019, 15, 1539. 30871407 10.1080/15548627.2019.1586248 PMC6693456

[30] E. Bahar , H. Kim , H. Yoon , Int. J. Mol. Sci. 2016, 17, 1558. 10.3390/ijms17091558 PMC5037829 27649160

[31] S. Dolai , S. Pal , R. K. Yadav , S. Adak , J. Biol. Chem. 2011, 286, 13638. 21330370 10.1074/jbc.M110.201889 PMC3075708

[32] M. Cirone , M. S. Gilardini Montani , M. Granato , A. Garufi , A. Faggioni , G. D'Orazi , J. Exp. Clin. Cancer Res. 2019, 38, 262. 31200739 10.1186/s13046-019-1275-z PMC6570888

[33] O. Kepp , L. Senovilla , I. Vitale , E. Vacchelli , S. Adjemian , P. Agostinis , L. Apetoh , F. Aranda , V. Barnaba , N. Bloy , Oncoimmunology 2014, 3, e955691.25941621

[34] a) H. Meng , A. E. Nel , Adv. Drug Delivery Rev. 2018, 130, 50; 10.1016/j.addr.2018.06.014 PMC6554022 29958925

[35] D. C. Drummond , C. O. Noble , Z. X. Guo , K. Hong , J. W. Park , D. B. Kirpotin , Cancer Res. 2006, 66, 3271. 16540680 10.1158/0008-5472.CAN-05-4007

[36] K. P. Olive , M. A. Jacobetz , C. J. Davidson , A. Gopinathan , D. McIntyre , D. Honess , B. Madhu , M. A. Goldgraben , M. E. Caldwell , D. Allard , K. K. Frese , G. Denicola , C. Feig , C. Combs , S. P. Winter , H. Ireland‐Zecchini , S. Reichelt , W. J. Howat , A. Chang , M. Dhara , L. Wang , F. Ruckert , R. Grutzmann , C. Pilarsky , K. Izeradjene , S. R. Hingorani , P. Huang , S. E. Davies , W. Plunkett , M. Egorin , R. H. Hruban , N. Whitebread , K. McGovern , J. Adams , C. Iacobuzio‐Donahue , J. Griffiths , D. A. Tuveson , Science 2009, 324, 1457. 19460966 10.1126/science.1171362 PMC2998180

[37] P. Tardi , S. Johnstone , N. Harasym , S. Xie , T. Harasym , N. Zisman , P. Harvie , D. Bermudes , L. Mayer , Leuk. Res. 2009, 33, 129. 18676016 10.1016/j.leukres.2008.06.028

[38] J. W. Wojtkowiak , J. M. Rothberg , V. Kumar , K. J. Schramm , E. Haller , J. B. Proemsey , M. C. Lloyd , B. F. Sloane , R. J. Gillies , Cancer Res. 2012, 72, 3938. 22719070 10.1158/0008-5472.CAN-11-3881 PMC3749826

[39] a) M. Femke , A. K. Goey , R. H. van Schaik , R. H. Mathijssen , S. Bins , Clin. Pharmacokinet. 2018, 57, 1229; 29520731 10.1007/s40262-018-0644-7 PMC6132501

[40] R. H. Mathijssen , R. J. van Alphen , J. Verweij , W. J. Loos , K. Nooter , G. Stoter , A. Sparreboom , Clin. Cancer Res. 2001, 7, 2182. 11489791

[41] S. John , J. Mls , M. Cervinka , E. Rudolf , Anti‐Cancer Agents Med. Chem. 2013, 13, 811. 10.2174/1871520611313050015 22721392

[42] a) C. De Duve , T. De Barsy , B. Poole , A. Trouet , P. Tulkens , F. o. Van Hoof , Biochem. Pharmacol. 1974, 23, 2495; 4606365 10.1016/0006-2952(74)90174-9

[43] A. C. Allison , M. R. Young , Life Sci. 1964, 3, 1407. 10.1016/0024-3205(64)90082-7 14248630

[44] C. A. Homewood , D. C. Warhurst , W. Peters , V. C. Baggaley , Nature 1972, 235, 50. 4550396 10.1038/235050a0

[45] a) T. Iwai , M. Sugimoto , D. Wakita , K. Yorozu , M. Kurasawa , K. Yamamoto , Oncotarget 2018, 9, 31411; 30140379 10.18632/oncotarget.25830 PMC6101148

[46] B. Bockorny , V. Semenisty , T. Macarulla , E. Borazanci , B. M. Wolpin , S. M. Stemmer , T. Golan , R. Geva , M. J. Borad , K. S. Pedersen , J. O. Park , R. A. Ramirez , D. G. Abad , J. Feliu , A. Munoz , M. Ponz‐Sarvise , A. Peled , T. M. Lustig , O. Bohana‐Kashtan , S. M. Shaw , E. Sorani , M. Chaney , S. Kadosh , A. Vainstein Haras , D. D. Von Hoff , M. Hidalgo , Nat. Med. 2020, 26, 878. 32451495 10.1038/s41591-020-0880-x

[47] a) T. Verfaillie , N. Rubio , A. D. Garg , G. Bultynck , R. Rizzuto , J. P. Decuypere , J. Piette , C. Linehan , S. Gupta , A. Samali , P. Agostinis , Cell Death Differ. 2012, 19, 1880; 22705852 10.1038/cdd.2012.74 PMC3469056

[48] D. Y. Wong , W. W. Ong , W. H. Ang , Angew. Chem., Int. Ed. 2015, 54, 6483. 10.1002/anie.201500934 25873535

[49] a) E. R. Lutz , A. A. Wu , E. Bigelow , R. Sharma , G. Mo , K. Soares , S. Solt , A. Dorman , A. Wamwea , A. Yager , Cancer Immunol. Res. 2014, 2, 616; 24942756 10.1158/2326-6066.CIR-14-0027 PMC4082460

[50] a) S. Yasmin‐Karim , P. T. Bruck , M. Moreau , S. Kunjachan , G. Z. Chen , R. Kumar , S. Grabow , S. K. Dougan , W. Ngwa , Front. Immunol. 2018, 9, 2030; 30245691 10.3389/fimmu.2018.02030 PMC6137176

[51] M. R. Kano , Y. Bae , C. Iwata , Y. Morishita , M. Yashiro , M. Oka , T. Fujii , A. Komuro , K. Kiyono , M. Kaminishi , Proc. Natl. Acad. Sci. USA 2007, 104, 3460. 17307870 10.1073/pnas.0611660104 PMC1800736

[52] C. Feig , J. O. Jones , M. Kraman , R. J. Wells , A. Deonarine , D. S. Chan , C. M. Connell , E. W. Roberts , Q. Zhao , O. L. Caballero , S. A. Teichmann , T. Janowitz , D. I. Jodrell , D. A. Tuveson , D. T. Fearon , Proc. Natl. Acad. Sci. USA 2013, 110, 20212. 24277834 10.1073/pnas.1320318110 PMC3864274

[53] J. A. Burger , A. Peled , Leukemia 2009, 23, 43. 18987663 10.1038/leu.2008.299

[54] a) S. T. Wang , H. P. Deng , P. Huang , P. Sun , X. H. Huang , Y. Su , X. Y. Zhu , J. Shen , D. Y. Yan , RSC Adv. 2016, 6, 12472;

