# Supplementary material for: Combination Chemo‐Immunotherapy for Pancreatic Cancer Using the Immunogenic Effects of an Irinotecan Silicasome Nanocarrier Plus Anti‐PD‐1
Source: Adv Sci (Weinh). 2021 Jan 27;8(6):2002147. doi: 10.1002/advs.202002147 (PMC7967046; doi:10.1002/advs.202002147)
Supplement: Supplementary file 1 — Supporting Information [file ADVS-8-2002147-s001.pdf]

© 2021 Wiley-VCH GmbH

ADVANCED SCIENCE
Open Access

Supporting Information

for *Adv. Sci.*, DOI: 10.1002/advs.202002147

Combination Chemo-Immunotherapy for Pancreatic Cancer Using the Immunogenic Effects of an Irinotecan Silicasome Nanocarrier plus Anti-PD-1

Xiangsheng Liu[1,2,3], Jinhong Jiang[2], Yu-Pei Liao[1], Ivanna Tang[1], Emily Zheng[1], Waveley Qiu[1], Matthew Lin[1], Xiang Wang[1,2], Ying Ji[1], Kuo-Ching Mei[1], Qi Liu[1], Chong Hyun Chang[2], Zev A. Wainberg[4], Andre E. Nel[1,2] and Huan Meng[1,2*]*

Supporting Information

Combination Chemo-Immunotherapy for Pancreatic Cancer Using the Immunogenic Effects of an Irinotecan Silicasome Nanocarrier plus Anti-PD-1

Xiangsheng Liu[1,2,3], Jinhong Jiang[2], Yu-Pei Liao[1], Ivanna Tang[1], Emily Zheng[1], Waveley Qiu[1], Matthew Lin[1], Xiang Wang[1,2], Ying Ji[1], Kuo-Ching Mei[1], Qi Liu[1], Chong Hyun Chang[2], Zev A. Wainberg[4], Andre E. Nel[1,2]* and Huan Meng[1,2]*

[1] Division of Nanomedicine, Department of Medicine, University of California, Los Angeles, California 90095, United States

[2] California NanoSystems Institute, University of California, Los Angeles, California 90095, United States

[3] The Cancer Hospital of the University of Chinese Academy of Sciences, Institute of Basic Medicine and Cancer (IBMC), Chinese Academy of Sciences, Hangzhou, Zhejiang 310022, China

[4] Division of Hematology Oncology, Department of Medicine, University of California, Los Angeles, California 90095, United States

*To whom correspondence should be addressed:

menghuan@g.ucla.edu or anel@mednet.ucla.edu

Fig. S1. Schematic to show the synthesis steps towards the production of a large batch of IRIN-loaded silicasome nanoparticles. These steps include: (1) synthesis of bare mesoporous silica nanoparticles (MSNP) synthesis at 20 L scale, using a sol-gel reaction as previously described[1]; (2) lipid coating after soaking in the trapping agent triethylammonium sucrose octasulfate (TEA_8SOS), through the use of continuous flow cell sonication procedure; and (3) remote loading of irinotecan, followed by column purification and sterilization. Box 1: An in-house equipment setup of the flow cell sonication system. Box 2: Schematic to show the mechanism of irinotecan remote loading. Silicasome particles containing the trapping agent were incubated in an IRIN solution, allowing the amphipathic drug to diffuse across the lipid bilayer. Proton release from the trapping agent converted the encapsulated IRIN to a hydrophilic derivative that cannot back-diffuse across the LB. The protonated drug interacts with negatively charged SOS^{8-} to form a drug precipitate[1,2].

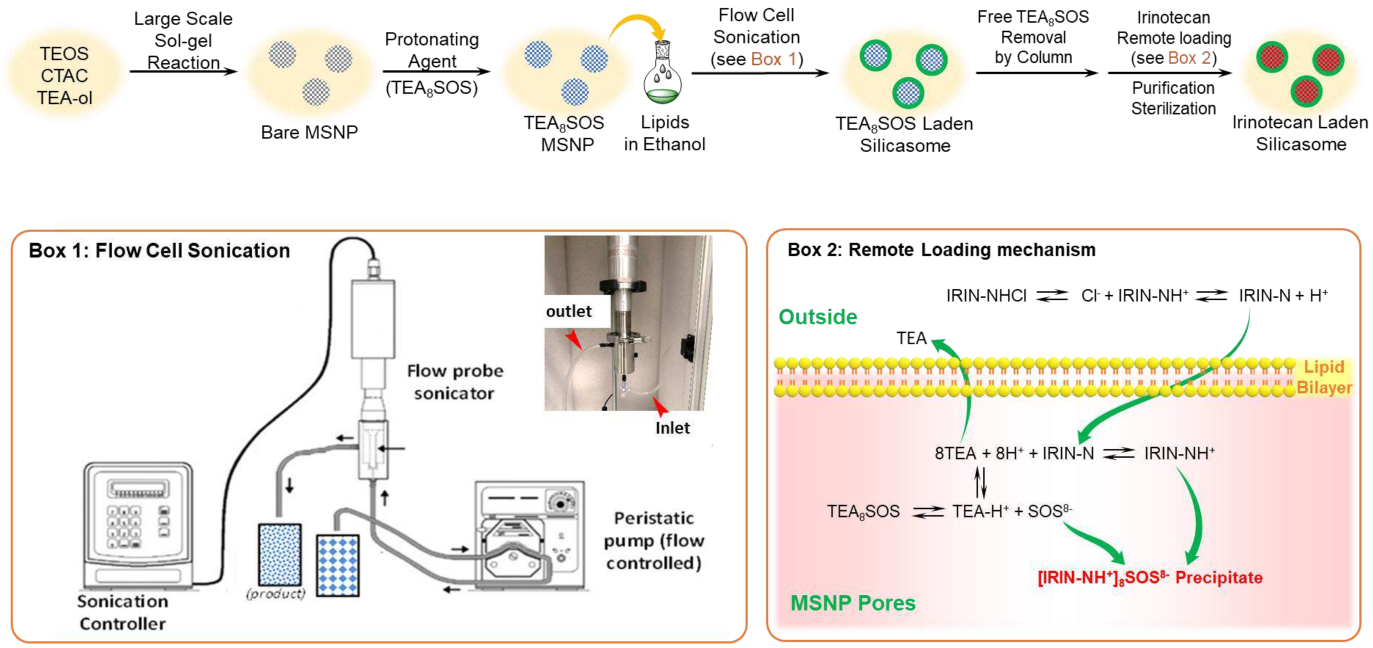

Fig. S2 Dose- and time-dependent study on IRIN-induced lysosomal alkalization effect in KPC cells. (A) Dose-dependence. KPC cells were treated with indicated free IRIN dose for 24 hours, followed by DND 99 and Hoechst dye co-staining, similar to Fig. 1C in the main manuscript. We were able to discern a major signal drop at 20 µM. IRIN accumulation in the KPC cells is indicated by white arrows. (B) Time-dependence. KPC cells were treated using 300 µM free IRIN for indicated time period, followed by DND 99/Hoechst co-staining, similar to (A). We were able to observe a major DND 99 signal drop as early as 1 h post incubation. Quantification of DND 99 fluorescence intensity in (A) and (B) higher presented in Fig. 1D in the main manuscript. (C) Similar to the experiment described in Fig. 1C, KPC cells were exposed to IRIN (300 µM) or chloroquine (CQ, 32 µM) for 24 h. The disappearance of the red fluorescence staining in the acidifying organellar environment in IRIN- and CQ-treated cells is as a result of the alkalization effect of these weak-base molecules. Bars are 10 µm. (D) The Henderson-Hasselbalch (H-H) equation allows calculation of the % protonated drug at different pH values. Although 83.3% of IRIN molecules are protonated at pH 7.4, the equilibrium shifts to ~99% of the molecules at a lysosomal relevant pH ~4.5-5.5. This equilibrium shift is still sufficient for depleting acidic the available protons in the lysosome, allowing alkalization to occur. It is possible to achieve this effect with an IRIN dose as low as ~20 µM (A).

(A)

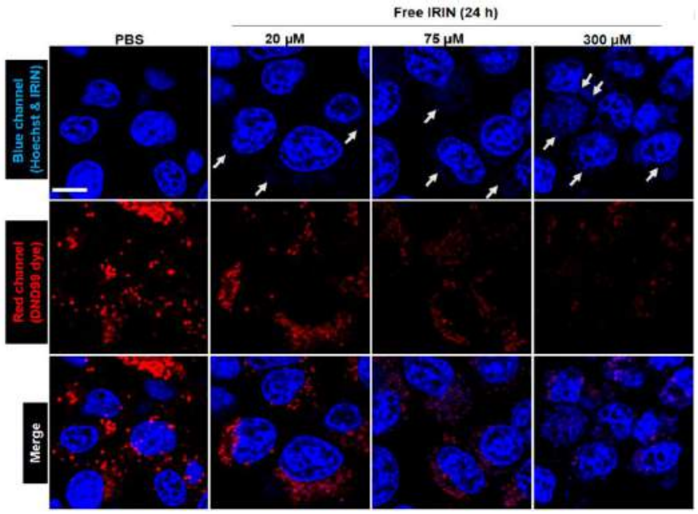

(B)

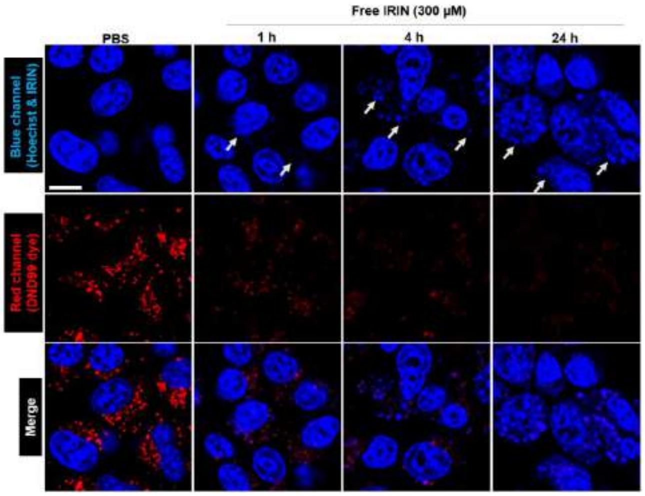

(C)

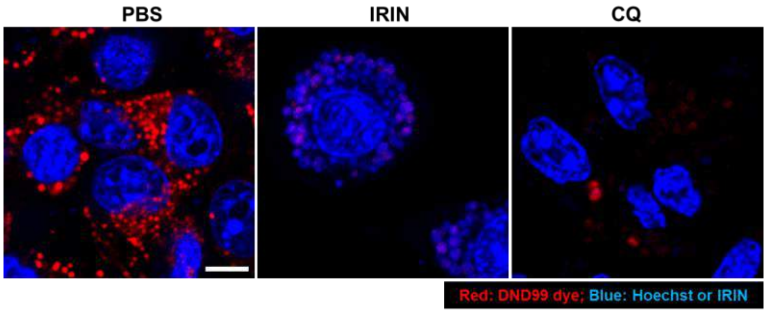

(D)

H-H equation: pH = pKa + log([A-]/[HA])

[A-]= concentration of an acid;

[HA] = concentration of conjugate base.

Fig. S3 Dose- and time-dependent study on IRIN induced changes on LC3B, p62 and PD-L1 in KPC cells. (A) KPC cells were treated with indicated free IRIN doses for 24 hours, followed by immunofluorescence (IF) staining, similar to Fig. 1E in the manuscript. (B) KPC cells were treated using 300 µM free IRIN for indicated time, followed by IF staining of LC3B, p62 and PD-L1. Bars represent 10 µm. Signal intensity was quantified by Image J software. At least three representative images were analyzed for each treatment. Data represents mean ± SD, n = 3. **p*<0.05, ***p*<0.01, ****p*<0.001 (1-way ANOVA followed by a Tukey's test).

(A)

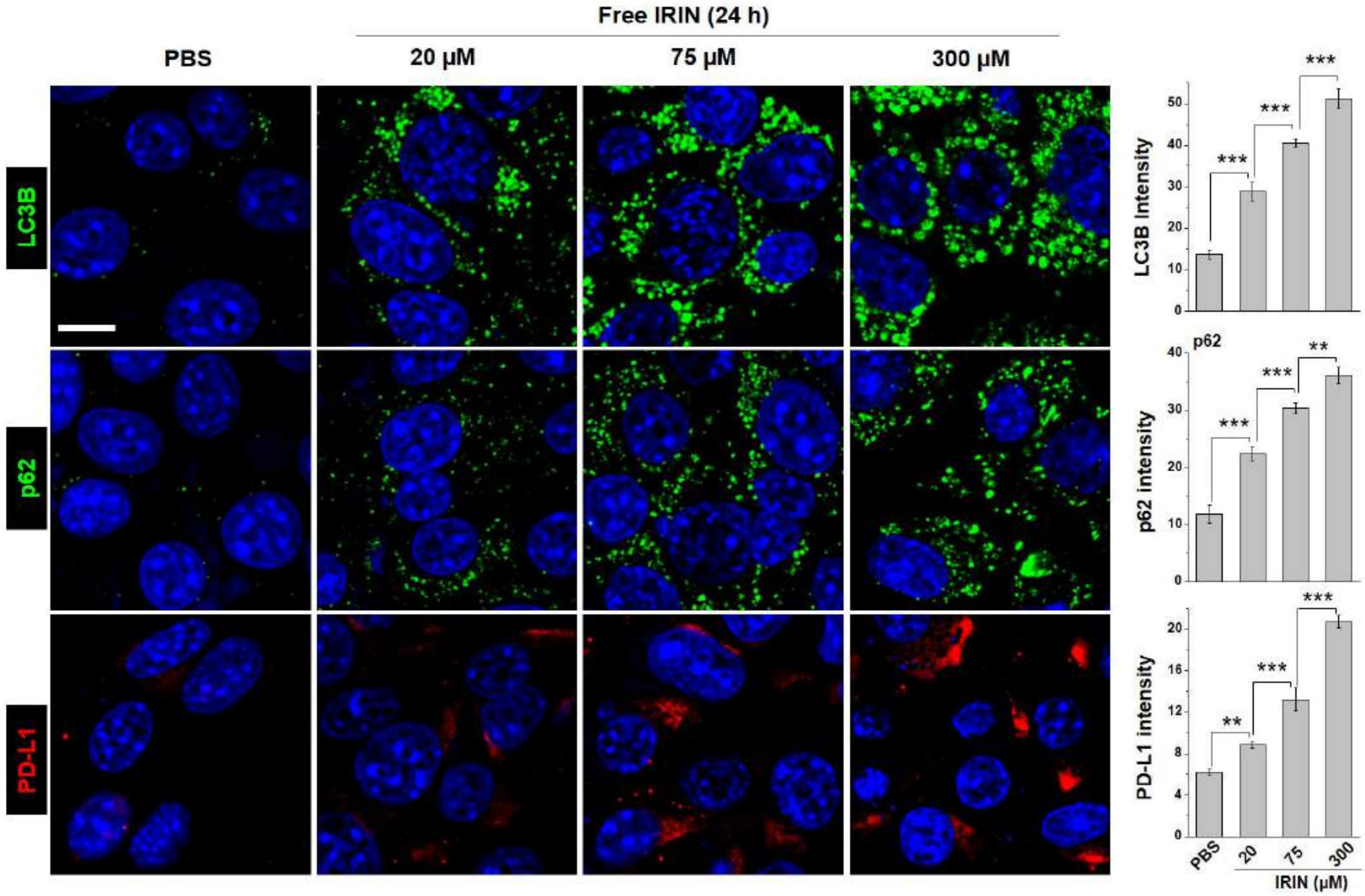

(B)

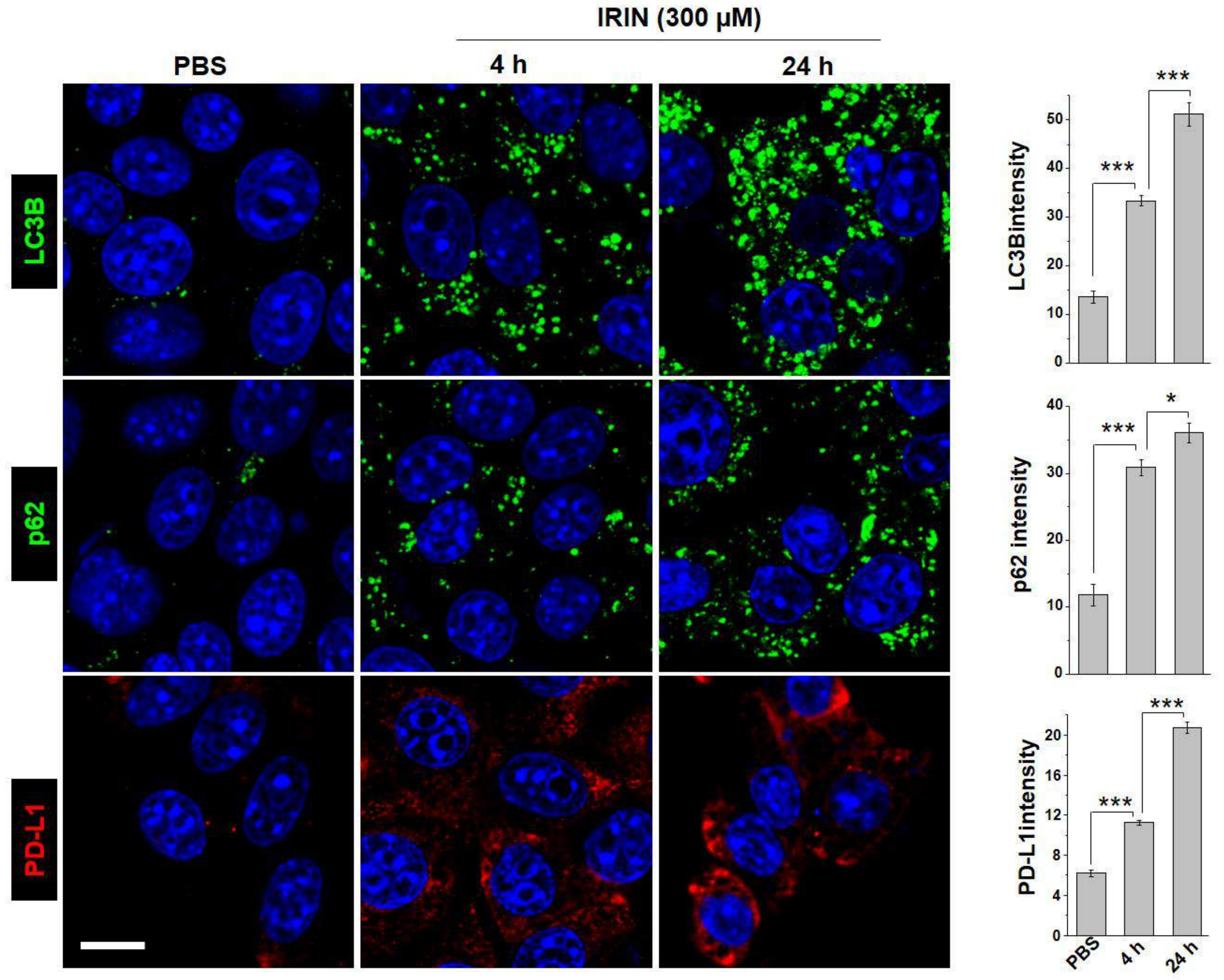
IRIN (300 µM)
PBS
4 h
24 h
LC3B
p62
PD-L1
LC3Bintensity
p62 intensity
PD-L1intensity
***
***
***
*
***
***
PBS
4 h
24 h

Fig. S4 IRIN treatment leads to the phosphorylation of the p65 NF-κB subunit in KPC cells. KPC cells were treated by free IRIN (300 μM) for 24 hrs, followed by immunoblotting assay for total and phosphorylated p65 NF-κB subunit. Vinculin was included to normalize the intensity of p-p65 abundance.

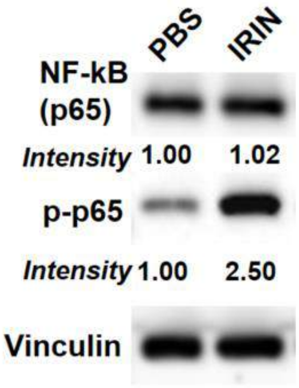

Fig. S5. Irinotecan, but not oxaliplatin, inhibited autophagy flux in KPC cells. KPC cells were treated with IRIN (300 µM) or oxaliplatin (OX, 500 µM) for 24 h. This was followed by a Western blotting analysis for expression of the autophagy markers, LC3 and p62. Densitometric analysis was performed by Image J software and the fold-intensity was normalized to that of housekeeping protein, β-Actin. Data represents mean ± SD, n=3. *p<0.05 compared to PBS group, #p<0.05 compared to OX group (1-way ANOVA followed by a Tukey's test).

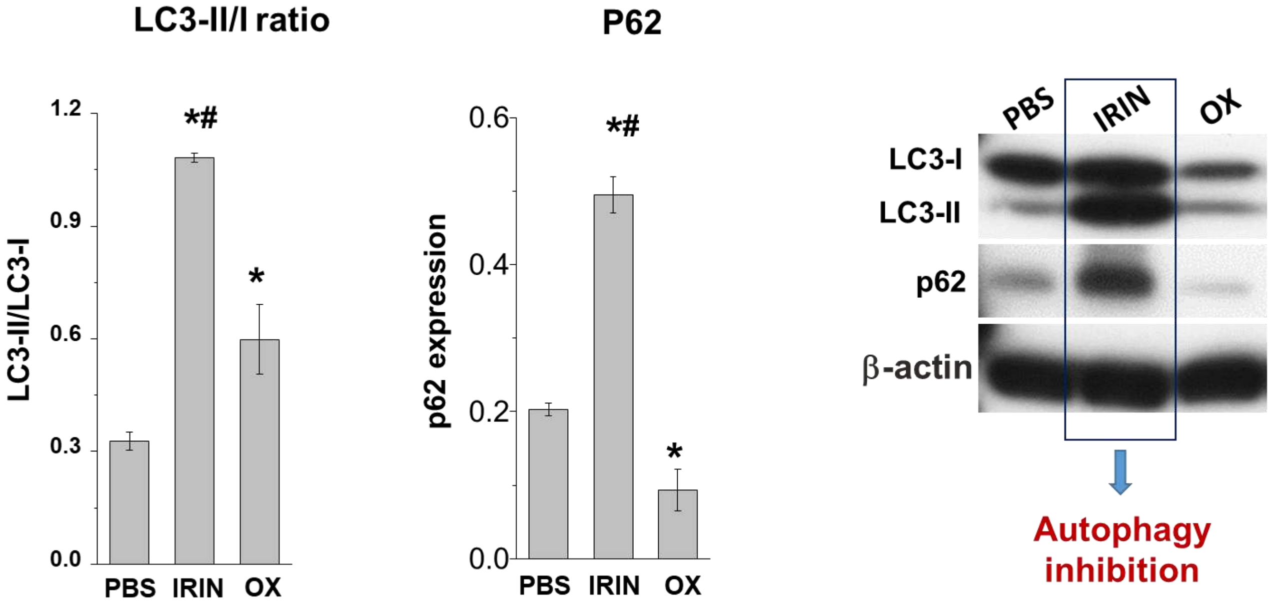

Fig. S6. Irinotecan, but not oxaliplatin, induced PD-L1 upregulation in KPC cells. KPC cells were treated with IRIN (300 µM) or OX (500 µM) for 24 h. PBS treatment was used as a negative control. PD-L1 expression was assayed using immunoblotting. Densitometric analysis was performed and normalized according to the expression levels of the housekeeping protein, vinculin. Data represents mean ± SD, n=3. *p<0.05 compared to PBS group, $^{\#}p$<0.05 compared to OX group (1-way ANOVA followed by a Tukey's test).

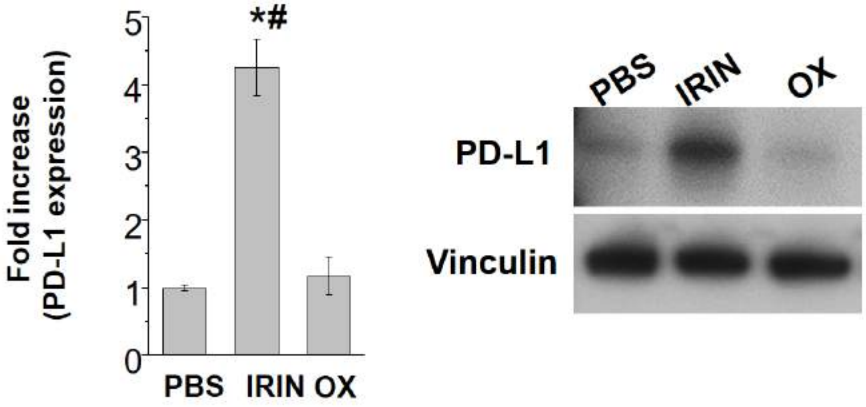

Fig. S7. Fluorescence microscopy to demonstrate the generation of reactive oxygen species (ROS) in KPC cells. Intracellular ROS production was determined by a fluorescence-based ROS Kit (Abcam, ab186027) in KPC cells. This assay measures total ROS species according to vendor's instructions. This assay is frequently used in cell biology to show the contribution of ROS in the context of ER stress and autophagy[3]. Briefly, KPC cells were treated with IRIN (300 µM), OX (500 µM) or TUN (10 µM) for 24 h. Images were captured using a fluorescence microscope. Signal intensity was quantified by Image J software. At least three representative images were analyzed for each treatment. Data represents mean ± SD, n=3. *p<0.05, ***p<0.001, compared to PBS group (1-way ANOVA followed by a Tukey's test). Bar is 50 µm.

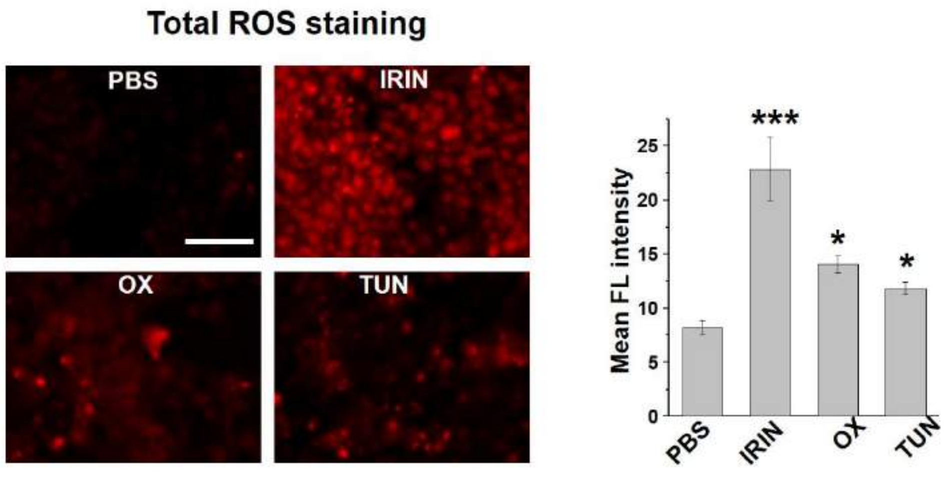

Fig. S8. Fluorescence microscopy to demonstrate intracellular Ca^{2+} release in KPC cells. Intracellular Ca^{2+} flux was assessed by a Fluo 4 AM assay in KPC cells. The cells were treated with IRIN (300 µM), OX (500 µM) or TUN (10 µM) for 24 h. Images were captured using a fluorescence microscope, followed by data analysis using the Image J software. At least three representative images were analyzed for each treatment. Data represents mean ± SD, n=3. ***p*<0.01, ****p*<0.001, compared to PBS group (1-way ANOVA followed by a Tukey's test). Bar is 50 µm.

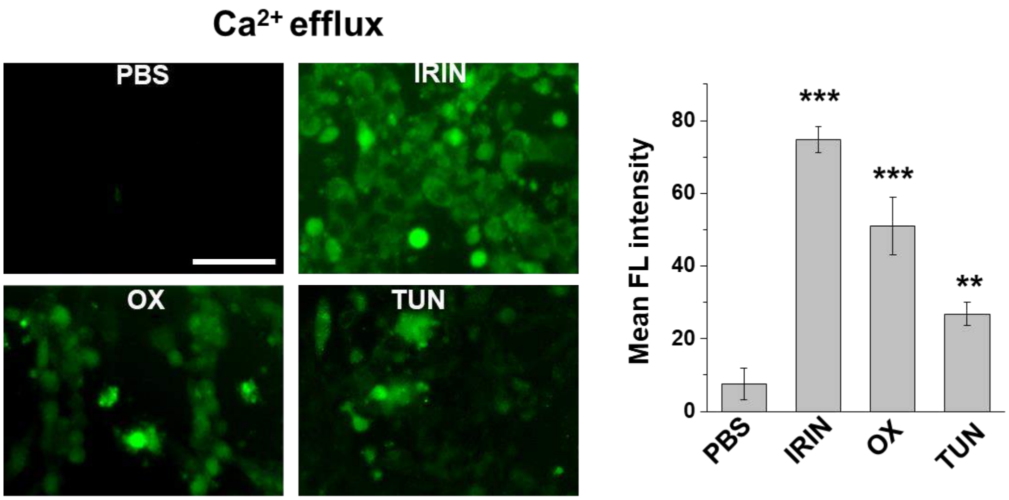

Fig. S9. Assessment of ecto-CRT expression in KPC cells. (A) Dose-dependent CRT expression in KPC cells treated by IRIN at different concentrations for 24 h. CRT expression was determined by flow cytometry as we described in the method section. Data represents mean ± SD (n = 3). *$p<0.05$, ***$p<0.001$ compared to PBS group (1-way ANOVA followed by a Tukey's test). (B) Confocal microscopy showing the appearance of CRT on the KPC cell surface of the cells treated with IRIN (300 μM) for 24 h. Bar is 20 μm. Green: CRT; Blue: Nuclear.

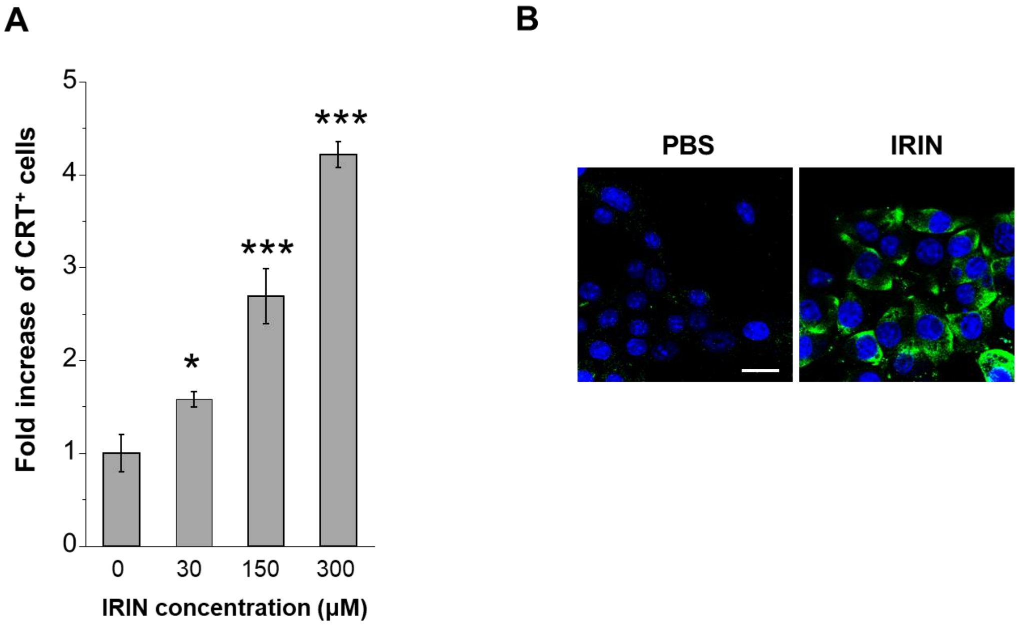

Fig. S10. IHC staining of CD8^{+} and FoxP3^{+} T cells in the vaccination experiment, as we described in the Fig. 2D in the main manuscript. Left panel: Quantification of CD8^{+} cytotoxic T cells and FoxP3^{+} regulatory T cells (Treg) from IHC staining. Data represents mean ± SEM (n=6), **p*<0.05, ***p*<0.01, ****p*<0.001 (1-way ANOVA followed by a Tukey's test). Right panel: Representative IHC images were shown. Bars are 50 µm. While there was no significant increase in CD8^{+} staining number, IRIN significantly (*p*<0.01) decreased FoxP3^{+} expression, which led to a significant increase of CD8^{+}/ FoxP3^{+} ratio as reflected in Fig. 2D.

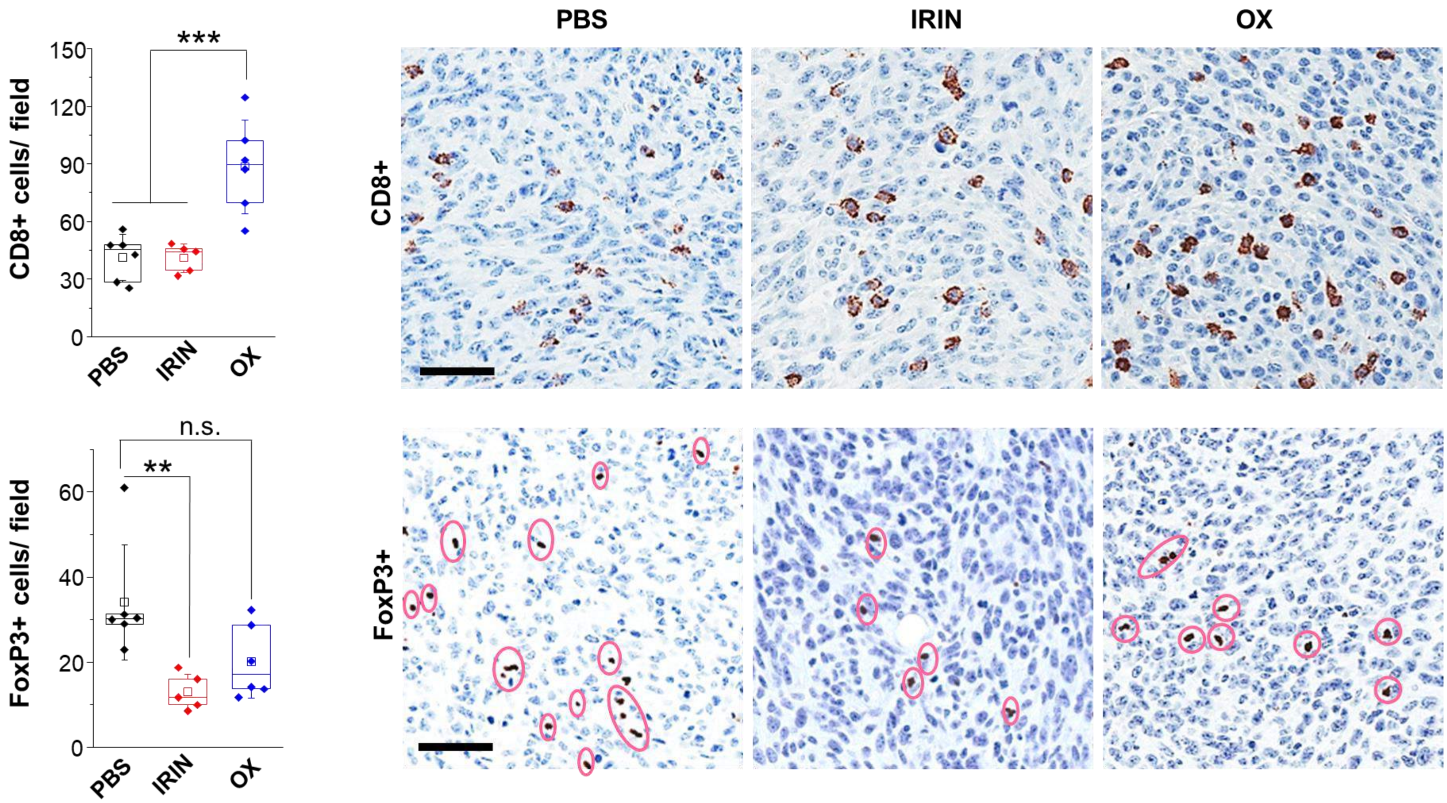

Fig. S11. IF staining of LC3B, p62 and PD-L1 in KPC cells treated with the silicasome w/wo IRIN. KPC cells were treated with IRIN silicasome at drug concentration of 300 µM for 24 hours. Empty silicasome (500 µg/mL, equivalent to drug dose of 300 µM) and PBS were used as control. IF staining of LC3B, p62 and PD-L1 was performed similar to Fig. S3. Bar is 10 µm.

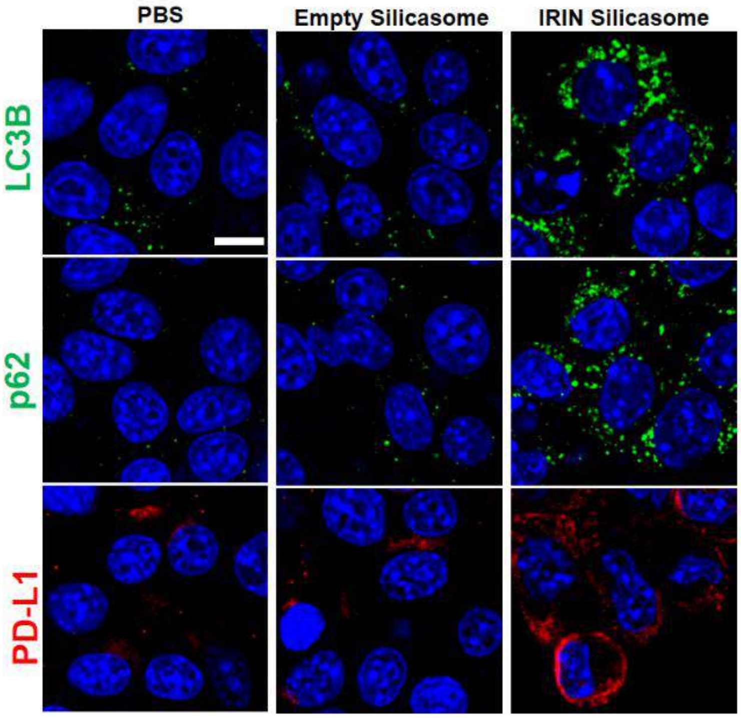

Fig. S12. IRIN silicasome induced ER stress in KPC cells. KPC cells were treated with IRIN silicasome at drug dose of 75 µM and 300 µM for 24 hours. PBS and empty silicasome (that is equivalent to drug dose of 300 µM) were included as control. Ca^{2+} efflux (A) and total ROS (B) measurements were performed similar to Figs. S7 and S8. Data represents mean ± SD, n=3. *p<0.05, **p<0.001, n.s., not significant (1-way ANOVA followed by a Tukey's test). (C) In a separate experiment, KPC cells were seeded in to a 6-well plate, followed by the treatment using silicasome w/wo IRIN (300 µM) for 48 hours. ER marker, CHOP protein, was assayed by western blotting similar to Fig. 2A in the main manuscript.

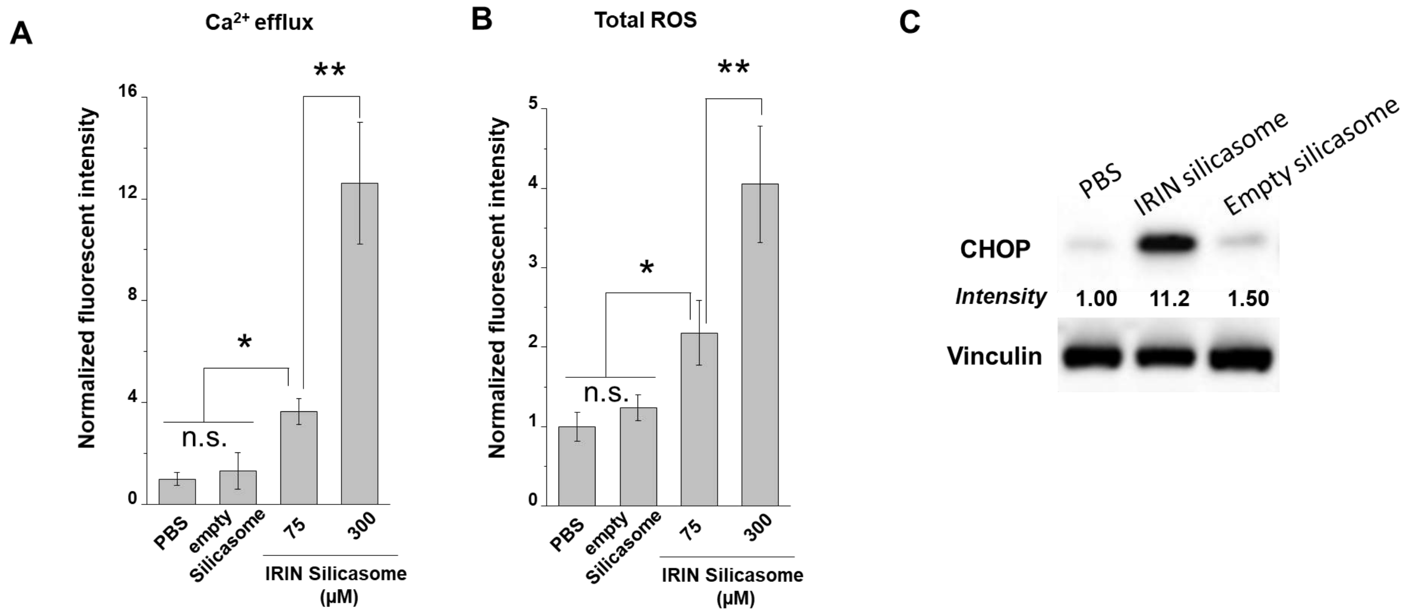

Fig. S13. Confirmative cellular studies in PANC-1 cells. To confirm the effect of IRIN, another PDAC cell line, i.e. PANC-1 cells, were used to study IRIN induced alkalization effect (A), autophagy inhibition (B) and PD-L1 induction. The treatments included free IRIN, IRIN silicasome and empty silicasome at equivalent drug concentration of 300 µM for 24 hrs. PBS served as a negative control. Bars: 10 µm. Signal intensity was quantified by Image J. At least three representative images were analyzed for each treatment. Data represents mean ± SD, n = 3. **p*<0.05, ***p*<0.01, ****p*<0.001 (1-way ANOVA followed by a Tukey's test). Consistent to the KPC data, both free and encapsulated IRIN were capable of neutralizing lysosomal pH, triggered LC-3B generation and increase PD-L1 expression in PANC-1 cells. No significant effect was observed when empty silicasome was tested.

(A)

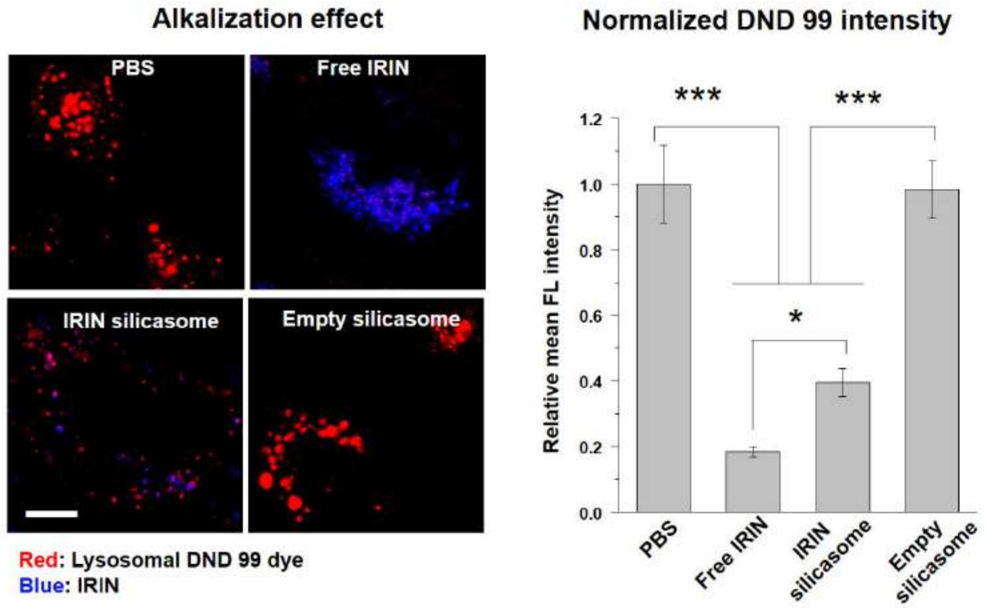

(B)

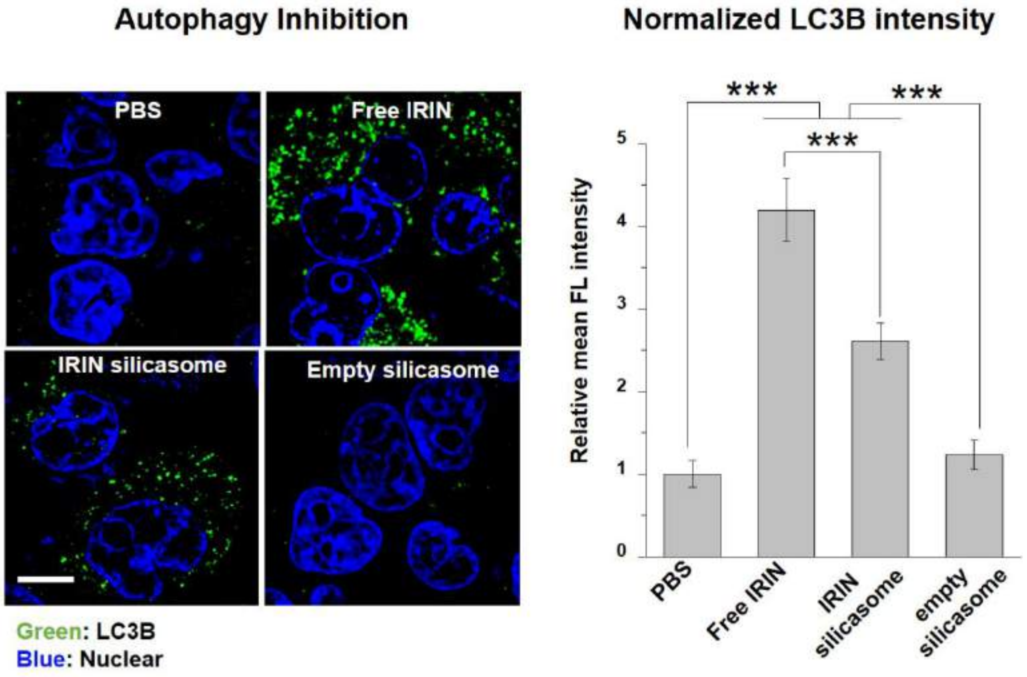

(C)

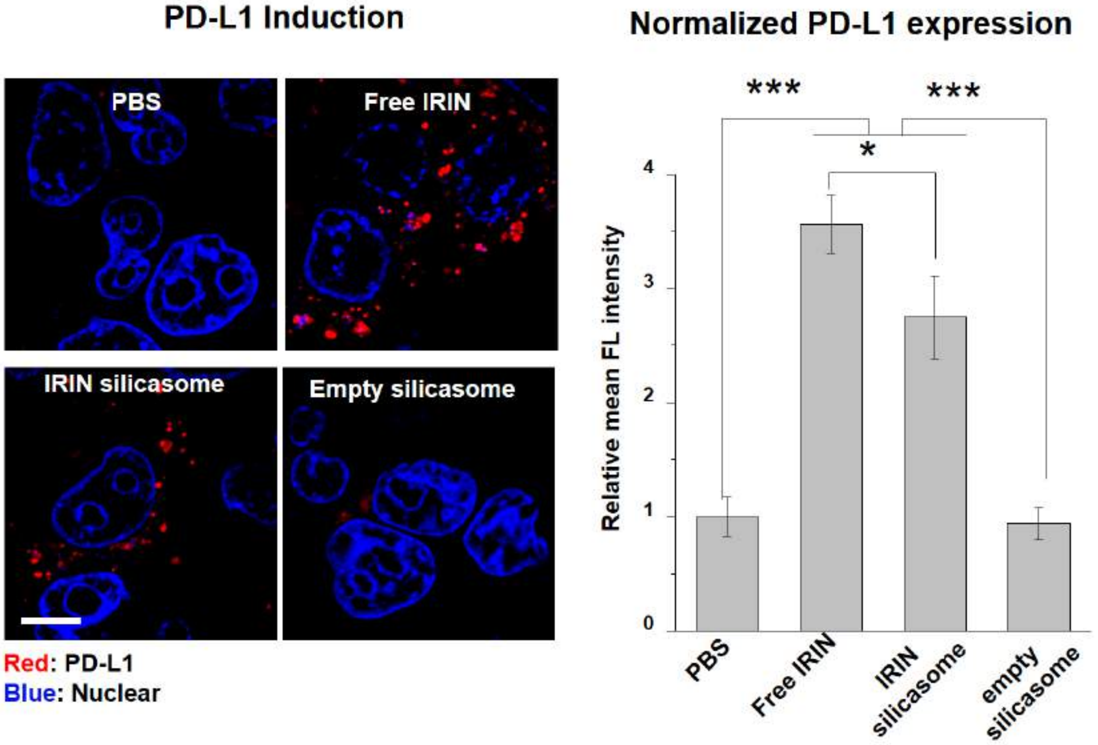
PD-L1 Induction
PBS
Free IRIN
IRIN silicasome
Empty silicasome
Red: PD-L1
Blue: Nuclear
Normalized PD-L1 expression
***
***
*
Relative mean FL intensity
0
1
2
3
4
PBS
Free IRIN
IRIN silicasome
empty silicasome

Fig. S14. Tumor weight measurement for the efficacy study shown in Fig. 5A. The animal treatment is described in the main manuscript. In addition to the IVIS imaging and ROI analysis that were described in the main manuscript, the excised tumor tissues were weighed at the time of sacrifice. Data represents mean ± SEM (n=3), *p<0.05.

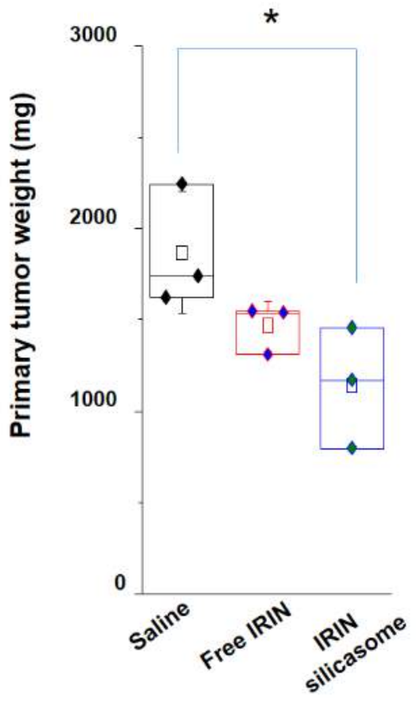

Fig. S15. HMGB1 staining and software assisted quantification of the amount of HMGB1 released from the nucleus. (**A**) Use of Aperio ImageScope software to quantify HMGB1 release in tumor tissues receiving different treatments. High resolution HMGB1 IHC pictures were scanned, followed by a software mediated imaging analysis process, which can discern "pixel density" in the picture. While the strong positive pixel density comes from the nuclear region (non-released HMGB1), the weak- or mid-positive regions come from the released HMGB1. The % of HMGB1 release was calculated by [(weak-positive + mid-positive pixel counts) / (total positive pixel counts)] x 100%. (**B**) Representative IHC images of HMGB1 staining in each treatment groups (Fig. 5D). Bar is 50 μm.

A

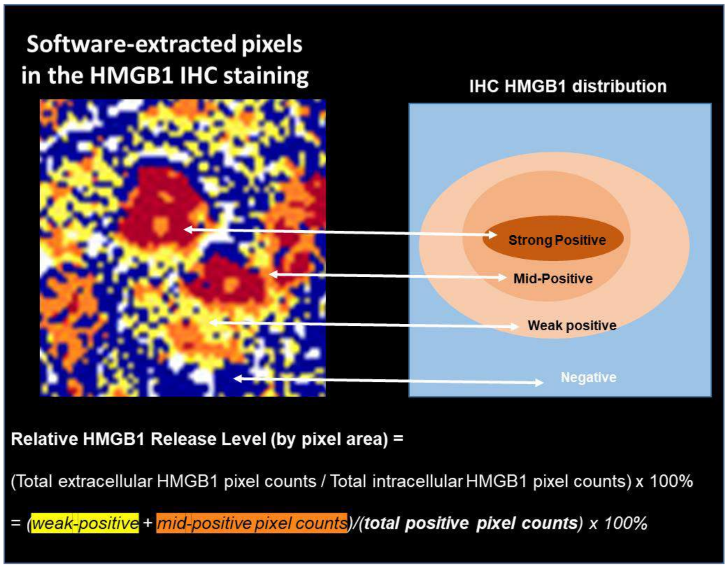

B

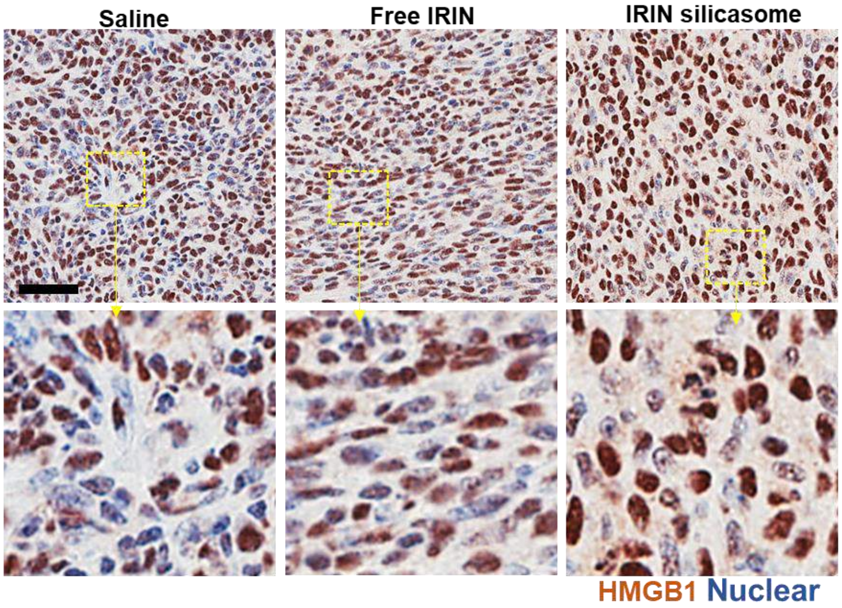

Fig. S16. IHC staining of CD8$^+$ and FoxP3$^+$ T cells in the efficacy experiment presented in the Fig. 5 of the main manuscript. Left panel: Quantification of CD8$^+$ cytotoxic T cells and FoxP3$^+$ Treg cells from the IHC staining. Data represents mean ± SEM (n=3), ***p*<0.01 (1-way ANOVA followed by a Tukey's test). Right panel: Representative IHC images were shown. Bars are 50 μm. While there was marginal effect of increasing CD8$^+$ number in response to IRIN silicasome, this treatment significantly decreased FoxP3$^+$ expression (p <0.01), which led to significant increase of CD8$^+$/ FoxP3$^+$ ratio as reflected in Fig. 6A.

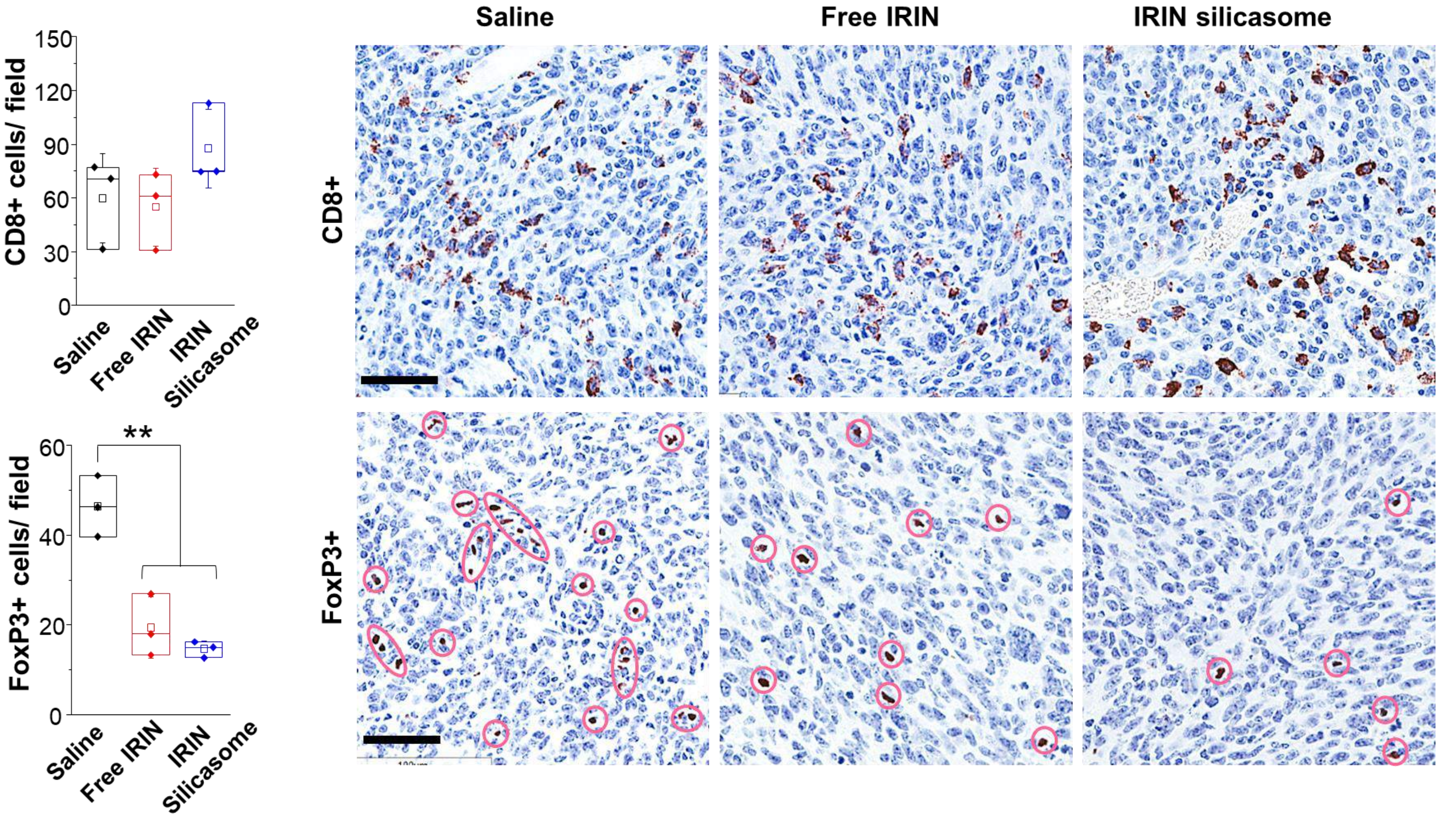

Fig. S17. Representative images of IHC staining of **(A)** perforin and granzyme, **(B)** IFN-γ and **(C)** PD-L1 in orthotopic KPC tumors in the efficacy experiment (Figs. 5 and 6). Bars are 50 µm.

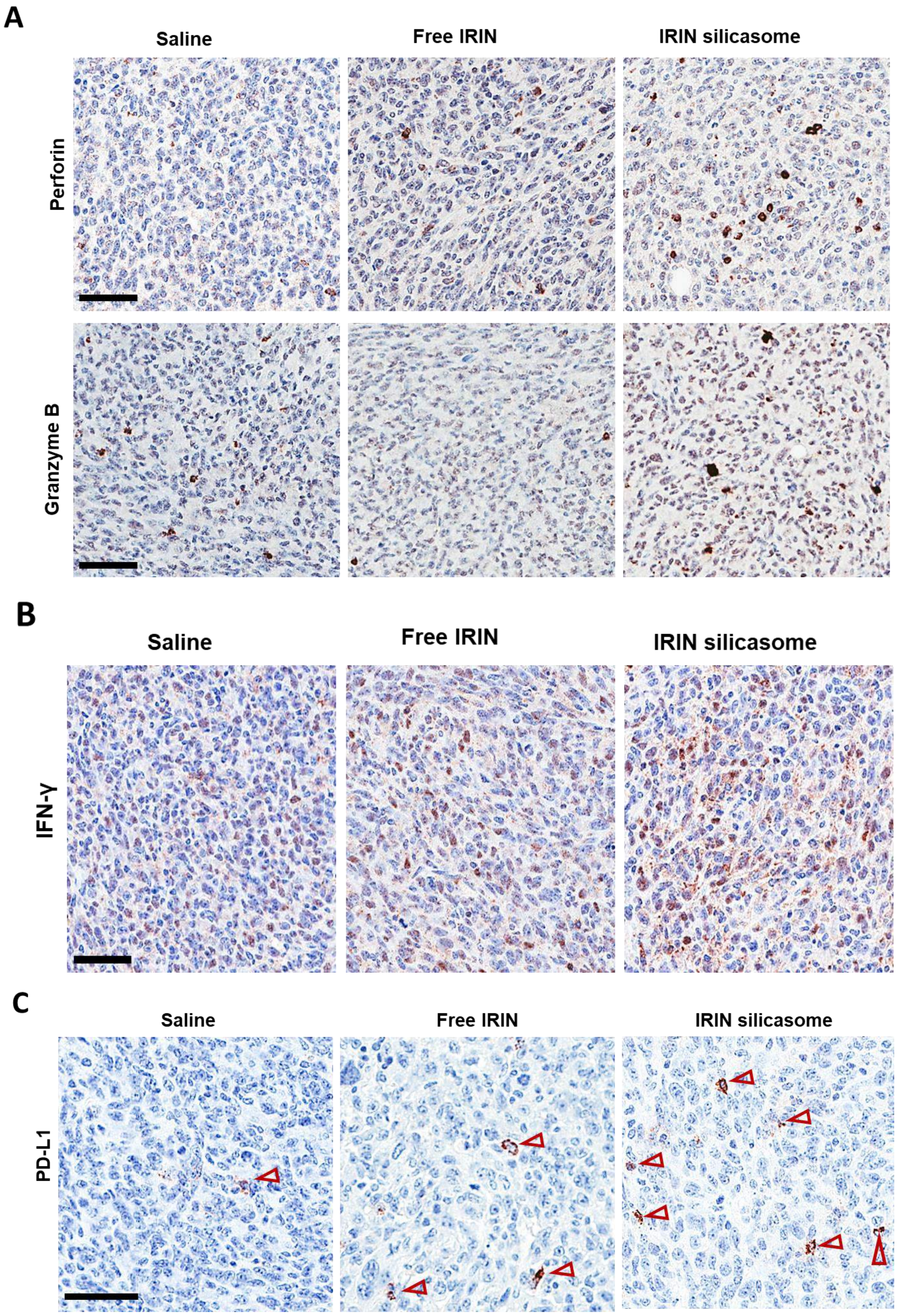

Fig. S18. Drug content at the orthotopic KPC tumor site after 24 h in animals receiving an IV injection of 40 mg/kg free irinotecan or IRIN silicasome. The irinotecan concentration was measured by LC-MS (Waters LCT Premier ESI). Data represent mean ± SD, N=3. ***p<0.001 by Student's t-test.

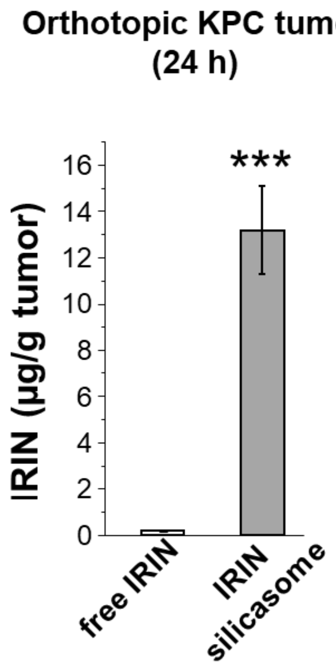

Fig. S19. IRIN release from silicasome at different pHs under abiotic conditions. IRIN laden silicasomes (100 µg/mL IRIN) were suspended in a 10 mM phosphonate buffer at pH 7.4 or pH 4.5, respectively. The suspension was incubated at 37 °C with shaking. At the indicated time period, 400 µL the particles suspension was centrifugated at 15 K rpm for 10 min, following which the released drug in the supernatant was analyzed through UV absorption at 360 nm. Data represent mean ± SD, n=3.

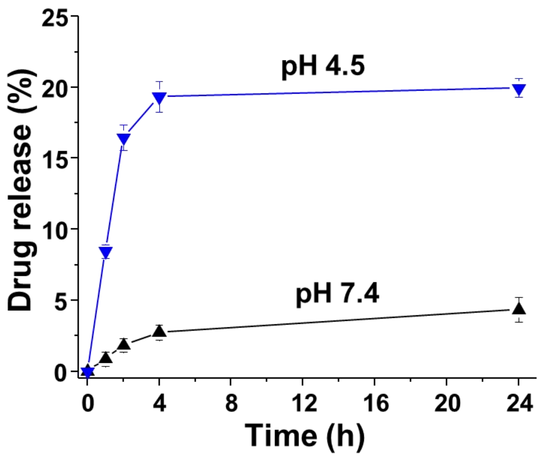

References:

(1) Liu, X.; Jiang, J.; Chan, R.; Ji, Y.; Lu, J.; Liao, Y. P.; Okene, M.; Lin, J.; Lin, P.; Chang, C. H.; Wang, X.; Tang, I.; Zheng, E.; Qiu, W.; Wainberg, Z. A.; Nel, A. E.; Meng, H. Improved Efficacy and Reduced Toxicity Using a Custom-Designed Irinotecan-Delivering Silicasome for Orthotopic Colon Cancer. *ACS Nano* **2019**, *13*, 38-53.

(2) Liu, X. S.; Situ, A.; Kang, Y. A.; Villabroza, K. R.; Liao, Y. P.; Chang, C. H.; Donahue, T.; Nel, A. E.; Meng, H. Irinotecan Delivery by Lipid-Coated Mesoporous Silica Nanoparticles Shows Improved Efficacy and Safety over Liposomes for Pancreatic Cancer. *Acs Nano* **2016**, *10*, 2702-2715.

(3) Yang, H.; Shen, H.; Li, J.; Guo, L. W. SIGMAR1/Sigma-1 receptor ablation impairs autophagosome clearance. *Autophagy* **2019**, *15*, 1539-1557.
